# *trans*‐Selective Insertional Dihydroboration of a *cis*‐Diborene: Synthesis of Linear sp^3^‐sp^2^‐sp^3^‐Triboranes and Subsequent Cationization

**DOI:** 10.1002/anie.201911645

**Published:** 2019-11-12

**Authors:** Uwe Schmidt, Luis Werner, Merle Arrowsmith, Andrea Deissenberger, Alexander Hermann, Alexander Hofmann, Stefan Ullrich, James D. Mattock, Alfredo Vargas, Holger Braunschweig

**Affiliations:** ^1^ Institut für Anorganische Chemie and Institute for Sustainable Chemistry & Catalysis with Boron Julius-Maximilians-Universität Wüzburg Am Hubland 97074 Würzburg Germany; ^2^ Department of Chemistry School of Life Sciences University of Sussex Brighton BN1 9QJ Sussex UK

**Keywords:** Cations, Diborenes, Hydroboration, Photoisomerization, Triboranes

## Abstract

The reaction of aryl‐ and amino(dihydro)boranes with dibora[2]ferrocenophane **1** leads to the formation 1,3‐*trans*‐dihydrotriboranes by formal hydrogenation and insertion of a borylene unit into the B=B bond. The aryltriborane derivatives undergo reversible photoisomerization to the *cis*‐1,2‐μ‐H‐3‐hydrotriboranes, while hydride abstraction affords cationic triboranes, which represent the first doubly base‐stabilized B_3_H_4_^+^ analogues.

## Graphical abstract

**A dibora[2]ferrocenophane** undergoes 100 % *trans*‐selective 1,2‐dihydroboration with amino‐ and aryldihydroboranes, with formal insertion of a borylene unit into the B=B double bond. The resulting aryltriboranes undergo reversible tautomerization under UV irradiation as well as hydride abstraction to the cationic H‐bridged triboranes.

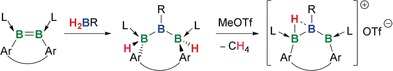

Unlike carbon, whose ability to form long and stable homonuclear chains is the basis of organic polymer chemistry, electron‐deficient boron has a strong tendency to oligomerize in the form of stable non‐classical clusters, in which three‐center‐two‐electron bonding predominates, especially within oligoboron hydrides.^1^ In contrast, classical oligoboranes of the form B_*n*_R_*n*+2_, in which each boron atom is sp^2^‐hybridized, are particularly prone to ligand scrambling and hydrolysis unless stabilized by electron‐donating amino or alkoxy substituents,^2^ as exemplified by the commercially available diboranes(4) B_2_(NMe_2_)_4_, B_2_Pin_2_ (Pin=pinacolato), B_2_Cat_2_ (Cat=catecholato), and B_2_Neop_2_ (Neop=neopentyl glycolato). In order to enforce electron‐precise B−B bonding in oligoboranes, therefore, Lewis bases are commonly used to electronically saturate the boron centers.^3^

The ubiquity of hydroborane and diborane reagents in organic synthesis^4^, ^5^ has fueled the search for new synthetic routes to a greater variety of electron‐precise di‐ and oligoboron hydrides. The reductive coupling of N‐heterocyclic carbene (NHC)‐stabilized (NHC)BX_2_R (R=Br, Ph) precursors, for example, provided access to neutral di‐ and tetrahydrodiboranes of the form [(NHC)R′HB‐BHR′(NHC)] (R′=H, Ph),^6^ whereas that of [ArBH_2_]_2_ diborane(6) precursors yielded [ArH_2_B‐BH_2_Ar]^2−^ dianions which were in turn converted via double hydride abstraction to neutral dihydrodiboranes(4).^7^ Milder routes to diboranes with terminal B−H bonds include the dehydrocoupling of boranes,^8^ selective dimethylamino‐hydride exchange at B_2_N_2_C_2_ heterocycles,^9^ the spontaneous transfer hydrogenation of diborenes with Me_2_NH⋅BH_3_,^10^ or the insertion of a borylene into a B−H bond at a boron cluster.^11^

Electron‐precise 1‐hydrotriboranes were obtained via the uncatalyzed hydroboration of 1,2‐diheteroaryldiborenes with HBCat (Scheme 1 a).^12^ Use of 9‐borabicyclo[3.3.1]nonane (9‐BBN) instead of HBCat led to a B_3_ *arachno* cluster, presumably due to the greater electron deficiency at boron in 9‐BBN.^13^ More recently, the double hydroboration of a diboryne to a 2,3‐dihydrotetraborane, followed by hydride abstraction, yielded the first cationic 2,3‐μ‐hydrotetraborane (Scheme 1 b).^14^ In this work we report a new strategy for the selective formation of doubly base‐stabilized *trans*‐1,3‐dihydrotriboranes by dihydroboration of a strained *cis*‐diborene, resulting in the formal hydrogenation of, and insertion of a borylene moiety into, the B=B bond. Furthermore, we study the photoisomerization and cationization of these species (Scheme 1 c).

**Scheme 1:**
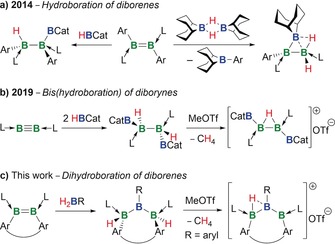
Atom‐efficient methods for the synthesis of electron‐precise oligoboron hydrides.

While studying the stoichiometric transfer hydrogenation of the ferrocene‐bridged diborene **1** 15 with Me_2_NH⋅BH_3_, we observed, beside the expected 1,2‐dihydrodiborane (δ_11B_=−18.0 ppm), a second product (δ_11B_=88.6, −29.6 ppm, 1:2 ratio), which we deemed to result from the reaction of **1** with the dehydrocoupling byproduct Me_2_N=BH_2_.^10^ Similarly, the reaction of **1** with 1 equiv pyrrolidinoborane (PyrBH_2_) in C_6_D_6_ at 60 °C overnight resulted in quantitative formation of the triborane **2‐Pyr** (Scheme 2), which shows two broad ^11^B NMR resonances at δ_11B_=87.8 (sp^2^‐*B*) and −28.8 (sp^3^‐*B*) ppm in a 1:2 ratio and a ^1^H{^11^B} NMR B*H* resonance (2 H) at δ_1H_=2.23 ppm.

**Scheme 2:**
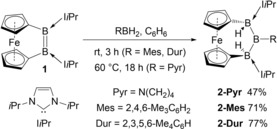
Addition of dihydroboranes to dibora[2]ferrocenophane **1**.

The analogous reactions of **1** with MesBH_2_ and DurBH_2_ (Mes=2,4,6‐Me_3_C_6_H_2_; Dur=2,3,5,6‐Me_4_C_6_H) yielded the triboranes **2‐Mes** and **2‐Dur** within three hours at room temperature (Scheme 2).^16^ **2‐Mes** presents two ^11^B NMR resonances in a 1:2 ratio at δ_11B_=100.6 and −13.6 ppm, similarly to **2‐Dur** at δ_11B_=107.6 and −14.7 ppm. These are significantly downfield‐shifted from **2‐Pyr** owing to the electron‐withdrawing nature of the aryl versus the electron‐donating nature of the amino substituent. Comparison with other literature‐known amino‐ and aryl(diboryl)boranes (δ(R_2_N*B*(BX_2_)_2_)≈50–62 ppm,^2^, ^17^ δ(Ar*B*(BX_2_)_2_)≈70–85 ppm)^18^ shows that the central boron nuclei of **2‐R** are unusually deshielded, that is, particularly electron‐poor. This was confirmed by density functional theory (DFT) calculations at the OLYP/TZ2P level of theory on **2‐Mes** in the gas phase, which gave negative Hirshfeld charges of −0.093 for B1 and B3 and a positive charge of 0.050 for B2 (Figure 3).

X‐ray crystallographic analyses of **2‐R** show a 1,3‐*trans*‐dihydro‐2‐R‐tribora[3]ferrocenophane structure (Figure 1 a, Figure S31 in the Supporting Information).^19^ With only one diastereomer present in their NMR spectra, we conclude that the addition of RBH_2_ to **1** is 100 % diastereoselective for the 1,3‐*trans*‐dihydrotriboranes. The presence of the two boron‐bound hydrogen atoms was confirmed by IR bands attributable to terminal B‐H vibrations in the 2160 to 2200 cm^−1^ region. These are the first examples of sp^3^‐sp^2^‐sp^3^‐hybridized triboranes, previous examples of electron‐precise triboranes being limited to sp^2^‐sp^2^‐sp^2^ 2, 17, 18 or sp^3^‐sp^3^‐sp^2^ hybridization patterns.^11^, ^13^ Unlike Nöth's tris(aminoboryl)[3]ferrocenophane, in which the central B2 atom is tilted out of the B1B3Fe plane,^20^ the iron center and all three boron atoms of **2‐R** lie in the same plane. Owing to the release of strain from the insertion of the third boron atom, the tilt angle between the two Cp ligands (*α* **2‐Pyr** 2.3°; **2‐Mes** 7.7°; **2‐Dur** 7.3°) is noticeably smaller than in diborene **1** (*α* 16.1°).^15^ In **2‐Pyr** the electron‐donating pyrrolidino substituent leads to an elongation of the B1−B2 bond (1.756(4) Å) and widening of the B1‐B2‐B1′ bond angle (127.0(3)°) compared to **2‐Mes** (Avg(B1−B2/3) 1.729(3) Å; B1‐B2‐B3 118.72(17)°) and **2‐Dur** (B1−B2 1.724(2) Å; B1‐B2‐B1′ 119.78(17)°).

**Figure 1:**
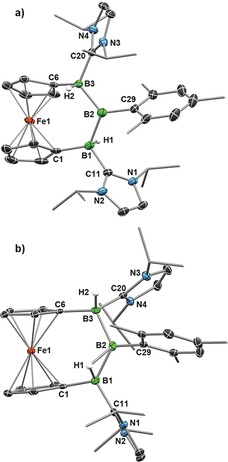
Crystallographically derived molecular structures of a) **2‐Mes** and b) **2′‐Mes**. Atomic displacement ellipsoids are set at 50 % probability. Ellipsoids of Me and *i*Pr groups and hydrogen atoms omitted for clarity except for boron‐bound hydrides.^19^, ^30^

Formally, the formation of **2‐R** involves the hydrogenation of and the insertion of the RB borylene unit into the B=B double bond of **1**. In contrast, the hydroboration of diborenes with HBCat proceeds by end‐on addition of the BCat unit to the diborene (Scheme 1 a).^11^ These new reactions therefore provide a complementary method of boron chain growth. Based on literature precedent, the reaction mechanism is likely to proceed via initial *syn*‐hydroboration of the diborene.^12^, ^14^ This would be followed by insertion of the RB fragment into the remaining B−B bond with concomitant migration of the second hydride to the terminal boron atom. DFT calculations show that the resulting *trans*‐1,3‐dihydrotriborane **2‐Mes** is favored over its *cis*‐isomer, **2′‐Mes**,^21^ by 2.35 kcal mol^−1^, accounting for the *trans*‐selectivity.

Solutions of **2‐Ar** in C_6_D_6_ were stable at 60 °C for 24 hours but when irradiated at room temperature for 18 hours two new ^11^B resonances appeared at δ_11B_=78.9 and −11.3 ppm (Ar=Mes) and δ_11B_=88.8 and −12.5 ppm (Ar=Dur), respectively (Scheme 3). Even with longer irradiation a maximum conversion of 75 % to the new species was achieved. The mixtures reverted back to **2‐Mes** and **2‐Dur** over several days at room temperature or overnight at 60 °C under the exclusion of light.^22^ In contrast, **2‐Pyr**, bearing an electronically stabilizing amino group, remained unchanged under irradiation.

**Scheme 3:**
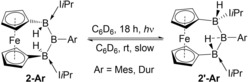
Reversible photoisomerization of **2‐Mes** and **2‐Dur**.

X‐ray diffraction analysis of single crystals obtained from a freshly irradiated solution of **2‐Mes** (Figure 1 b)^23^ revealed the structure of the *cis*‐isomer **2′‐Mes**, in which H1 has shifted from a terminal position *trans* to H2 to a bridging position *cis* to the terminal H2.^19^ This is accompanied by a shortening of the B1−B2 bond from 1.720(3) to 1.650(3) Å, a lengthening of the B2−B3 bond from 1.738(3) to 1.774(3) Å and a slight widening of the B1‐B2‐B3 angle from 118.72(17) to 122.69(16)°. Furthermore, the Fe atom no longer lies in the B_3_ plane.

Despite their unsymmetrical solid‐state structure, **2′‐Mes** and **2′‐Dur** show only one ^11^B NMR resonance and a single ^1^H{^11^B} NMR B*H* resonance integrating for 2H around 2.6 ppm in solution. Since a *cis*‐isomer with two terminal B−H bonds can be ruled out by computations, we propose that in solution H1 and H2 undergo rapid bridging/terminal exchange, leading to the apparent symmetry.

Optimization of a low‐lying excited state of **2‐Mes** provides insight into a possible mechanism for the tautomerization. Indeed, starting from the *trans*‐geometry of **2‐Mes**, the system smoothly adopts the same structural characteristics as **2′‐Mes**, that is, one bridging and one terminal hydride, albeit in a *trans* configuration. Migration of the bridging hydride to the *cis* position then proceeds with transient breaking and reforming of the B1−B2 bond (see the Supporting Information for details).

DFT calculations on **2′‐Mes** yield Hirshfeld charges of −0.059 for B1, −0.018 for B2, and −0.090 for B3 (Figure 3), which reflect the charge flux established between B1 and B2 through the bridging of H1. Furthermore, H1 has lost its hydridic character (−0.005), whereas the terminal H2 has become more hydridic (−0.074).

The increased hydricity of H2 prompted us to attempt its selective abstraction. The addition of methyl triflate (MeOTf) to **2‐Ar** resulted in the abstraction of one hydride and quantitative formation of the cationic triboranes **3‐Ar** (Scheme 4).^24^ The ^11^B NMR spectra of **3‐Mes** and **3‐Dur** display three distinct, broad 1:1:1 resonances around 80, 46 and 20 ppm. The complex ^1^H NMR spectra are indicative of highly unsymmetrical and/or geometrically constrained compounds. The broad ^1^H{^11^B} NMR B*H* resonances at 0.63 (**3‐Mes**) and 0.81 ppm (**3‐Dur**) are significantly upfield‐shifted from those of **2‐Ar** (ca. 2.9 ppm) and **2′‐Ar** (ca. 2.6 ppm). Furthermore, the IR spectra of **3‐Ar** are free of the terminal B‐H vibration bands displayed by **2‐Ar**, but show bands in the 1560–1570 cm^−1^ region attributable to bridging hydrides.^25^ Unlike **2′‐Ar**, **3‐Ar** show no fluxionality in solution up to 80 °C and remain unchanged under UV irradiation.

**Scheme 4:**
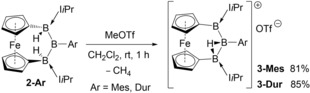
Cationization of **2‐Mes** and **2‐Dur** by hydride abstraction.

Single‐crystal X‐ray diffraction analyses of **3‐Mes** (Figure 2) and **3‐Dur** (see Figure S33 in the Supporting Information) confirmed their cationic 1,2‐μ‐hydro‐2‐aryltribora[3]ferrocenophane structures.^19^ While there have been recent reports of linear B_3_H_6_^−^ anions,^26^ and of cyclic doubly base‐stabilized B_3_H_6_^+^ cations,^25^ these are, to our knowledge, the first examples of linear triborane cations. As doubly base‐stabilized analogues of the B_3_H_4_^+^ cation they are also structurally related to the B_3_H_6_^−^ anion, for which ab initio studies predict a similar *C*_1_ symmetry, with a linear B_3_ unit containing a μ‐bridging hydride as the structural minimum.^27^ Interestingly, the B−B bonds lengths in **3‐Ar** are all near‐identical (1.658(2)–1.667(4) Å) and significantly shorter than those in **2‐Ar** (1.720(3)–1.738(3) Å), as is expected upon cationization. The B1‐B2‐B3 angle also narrows considerably from 122.69(16)° in **2′‐Mes** to ca. 111° in **3‐Ar**. Furthermore, the dip angle of the B3 moiety (ca. 17°) is significantly larger than that of the B1 moiety (ca. 7°). This leads to the B3⋅⋅⋅Fe distance (**3‐Mes** 2.910(2), **3‐Dur** 2.920(2) Å) being much shorter than the B1⋅⋅⋅Fe distance (**3‐Mes** 3.163(3), **3‐Dur** 3.149(2) Å) and is indicative of a through‐space interaction between the cationic B3 and electron‐rich Fe^II^ centers (Figure 3).^28^

**Figure 2:**
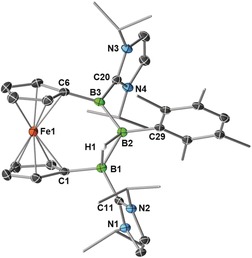
Crystallographically derived molecular structure of the **3‐Dur** cation. Thermal ellipsoids are set at 50 % probability. Thermal ellipsoids of Me and *i*Pr groups, the OTf^−^ counteranion and hydrogen atoms omitted for clarity except for boron‐bound hydrides.^19^, ^30^

**Figure 3:**
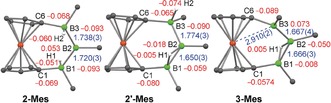
Solid‐state structures of **2‐Mes**, **2′‐Mes** and **3‐Mes** (I*i*Pr and Mes ligands omitted for clarity). Experimental bond lengths [Å] in blue, calculated Hirshfeld charges in red.

DFT calculations on **3‐Mes** give calculated Hirshfeld charges of −0.008 for B1, −0.050 for B2 and 0.073 for B3 (Figure 3). This enables the attribution of the three ^11^B NMR resonances as follows: δ(B1)=46, δ(B2)=20 and δ(B3)=80 ppm. A comparison with the partial charges calculated for **2′‐Mes** reveals a considerable change in charge density distribution upon abstraction of the terminal hydride at B3. Moreover, the bridging hydrogen H1 has now acquired a very small positive charge (+0.005), suggesting a slightly acidic character.

In conclusion, we have shown that the addition of dihydroboranes to a strained *cis*‐diborene provides a complementary method to the addition of monohydroboranes to diborenes for the formation of electron‐precise triboranes. The *trans*‐1,3‐dihydro‐2‐aryltriboranes undergo fully reversible phototautomerization as well as facile hydride abstraction to yield the first stable, doubly base‐stabilized analogues of the B_3_H_4_^+^ cation. X‐ray structural and DFT analyses reveal significant geometry and charge distribution fluctuations between the various B_3_ species. The flexibility of the ferrocenediyl‐bridged B_3_ core in easily accommodating (and giving up) charge should make these compounds particularly interesting for further reactivity studies.^29^

## Conflict of interest

The authors declare no conflict of interest.

## Supporting information

As a service to our authors and readers, this journal provides supporting information supplied by the authors. Such materials are peer reviewed and may be re‐organized for online delivery, but are not copy‐edited or typeset. Technical support issues arising from supporting information (other than missing files) should be addressed to the authors.

Supplementary
