# Supplementary material for: trans‐Selective Insertional Dihydroboration of a cis‐Diborene: Synthesis of Linear sp3‐sp2‐sp3‐Triboranes and Subsequent Cationization
Source: Angew Chem Int Ed Engl. 2019 Nov 12;59(1):325–9. doi: 10.1002/anie.201911645 (PMC6972689; doi:10.1002/anie.201911645)
Supplement: Supplementary file 1 — Supplementary [file ANIE-59-325-s001.pdf]

Angewandte Chemie
Eine Zeitschrift der Gesellschaft Deutscher Chemiker

Supporting Information

trans-Selective Insertional Dihydroboration of a *cis*-Diborene: Synthesis of Linear sp^3-sp^2-sp^3-Triboranes and Subsequent Cationization

*Uwe Schmidt, Luis Werner, Merle Arrowsmith, Andrea Deissenberger, Alexander Hermann, Alexander Hofmann, Stefan Ullrich, James D. Mattock, Alfredo Vargas, and Holger Braunschweig**

anie_201911645_sm_miscellaneous_information.pdf

Supporting Information

Table of contents

Methods and materials 2

Synthetic procedures 3

NMR spectra of isolated compounds 7

NMR spectra of irradiated samples 24

Kinetics of the thermal isomerization of **2'-Mes** back to **2-Mes** 28

Cationization of **2-Pyr** 31

IR spectra 32

X-ray crystallographic data 35

Computations 41

References 47

Methods and materials

All manipulations were performed either under an atmosphere of dry argon or *in vacuo* using standard Schlenk line or glovebox techniques. Deuterated solvents were dried over molecular sieves and degassed by three freeze-pump-thaw cycles prior to use. All other solvents were distilled and degassed from appropriate drying agents. Solvents were stored under argon over activated 4 Å molecular sieves. NMR spectra were acquired on a Bruker Avance 500 NMR spectrometer. Chemical shifts (δ) are provided in ppm and internally referenced to the carbon nuclei ($^{13}C\{^{1}H\}$) or residual protons (^{1}H) of the solvent. Heteronuclei NMR spectra are referenced to external standards (^{11}B: $BF_3{\cdot}OEt_2$; ^{19}F: Cl_3CF). Microanalyses (C, H, N, S) were performed on an Elementar vario MICRO cube elemental analyzer. High-resolution mass spectrometry data were obtained using a Thermo Scientific Exactive Plus spectrometer in LIFDI mode.

Solvents and reagents were purchased from Sigma-Aldrich or Alfa Aesar. The diboraferrocenophane **1**[1] and the dihydroboranes $MesBH_2$ (Mes = 2,4,6-$Me_3C_6H_2$) and $DurBH_2$ (Dur = 2,3,5,6-Me_4C_6H)[2] were synthesized following literature procedures.

Synthetic procedures

Synthesis of 2-Pyr

A mixture of **1** (96 mg, 188 μmol) and H_2B-Pyr (15.6 mg, 188 μmol) was stirred overnight at 60 °C in 10 mL benzene. The reaction mixture turned orange. Insoluble residues were removed by filtration and volatiles were removed under reduced pressure. The crude yellow product was washed with hexanes (3 x 5 mL) and recrystallized from THF to afford **2-Pyr** as a yellow crystalline solid (47 mg, 88 μmol, 47%). $^1H\{^{11}B\}$ NMR (500 MHz, C_6D_6, 297 K): δ = 6.29, 6.26 (two d, ^3J = 1.8 Hz, 2H each, NCH_{IiPr}), 5.72, 5.33 (two sept, ^3J = 6.7 Hz, 2H each, CH_{iPr}) 4.43, 4.40, 4.17 (three m, 2H each, CH_{Cp}), 3.71 (m, 2H, $NCH_{2\text{-Pyr}}$), 3.59 (m, 2H, CH_{Cp}), 2.42 (m, 2H, $NCH_{2\text{-Pyr}}$), 2.23 (s, 2H, B*H*), 1.61 (m, 4H, $NCH_2CH_{2\text{-Pyr}}$), 1.43, 1.35, 1.04, 0.87 (four d, ^3J = 6.7 Hz, 6H each, $CH_{3\text{-}iPr}$), ppm. $^{13}C\{^1H\}$ NMR (126 MHz, C_6D_6, 297 K): δ = 177.0 (br, $C_{carbene}$), 115.0 (NCH_{IiPr}), 114.1 (NCH_{IiPr}), 101.2 (br, $C_{q\text{-Cp}}$), 73.8, 72.8, 66.3, 65.9 (CH_{Cp}) 51.7 ($NCH_{2\text{-Pyr}}$), 50.1, 48.9 (CH_{iPr}), 27.7 ($NCH_2CH_{2\text{-Pyr}}$), 24.2, 23.7, 22.8, 22.0 ($CH_{3\text{-}iPr}$) ppm. ^{11}B NMR (160 MHz, C_6D_6, 297 K): δ = 87.7 (sp^2-*B*), –28.8 (sp^3-*B*) ppm. Solid-state IR: υ(B*H*) = 2202 (very broad) cm^{-1}. Elemental analysis (%) for $C_{32}H_{50}B_3FeN_4$ (M_w = 593.1): calcd. C 64.81, H 8.50, N 11.81; found: 64.97, H 8.59, N 11.17.

Synthesis of 2-Mes

A mixture of **1** (100 mg, 196 μmol) and H_2B-Mes (26 mg, 196 μmol) was stirred in 10 mL benzene for 3 h at room temperature. The reaction mixture turned yellow. Insoluble residues were removed by filtration, volatiles were removed under reduced pressure. The crude yellow product was washed with hexane (3 x 5 mL) and dried *in vacuo* to afford compound **2-Mes** as a yellow solid (89 mg, 139 μmol, 71%). $^1H\{^{11}B\}$ NMR (500 MHz, C_6D_6, 297 K): δ = 6.63 (s, 2H, CH_{Mes}), 6.09 (br, 4H, NCH_{IiPr}), 5.71, 4.75 (two br, 2H each, CH_{iPr}), 4.78, 4.61, 4.55, 4.35 (four m, 2H each, CH_{Cp}), 2.88 (s, 2H, B*H*), 2.29 (s, 6H, *o*-$CH_{3\text{-Mes}}$), 2.19 (s, 3H, *p*-$CH_{3\text{-Mes}}$), 1.04, 0.77, 0.50 (three br, 12H:6H:6H, $CH_{3\text{-IiPr}}$) ppm. $^{13}C\{^1H\}$ NMR (126 MHz, C_6D_6, 297 K): δ = 174.2 (br, $C_{carbene}$), 156.5 ($C_{q\text{-Mes}}$), 133.6 ($C_{q\text{-Mes}}$), 131.3 ($C_{q\text{-Mes}}$), 127.1 ($CH_{\text{-Mes}}$), 114.1 (NCH_{IiPr}), 114.0 (NCH_{IiPr}), 90.25 (br, $C_{q\text{-Cp}}$), 78.5, 77.7, 69.2, 68.8 (CH_{Cp}) 49.0, 48.5 (CH_{iPr}), 24.9, 23.6 ($CH_{3\text{-}iPr}$), 22.3 (*o*-$CH_{3\text{-Mes}}$), 21.8, 21.3 ($CH_{3\text{-}iPr}$) 21.2 (*p*-$CH_{3\text{-Mes}}$) ppm. ^{11}B NMR (160 MHz, C_6D_6, 297 K): δ = 100.6 (sp^2-*B*), –13.6 (sp^3-*B*) ppm. Solid-state IR: υ(B*H*) = 2204, 2182 cm^{-1}. Elemental analysis (%) for $C_{37}H_{53}B_3FeN_4$ (M_w = 642.2): calcd. C 69.21, H 8.32, N 8.73; found: C 69.16, H 8.28, N 8.41.

Synthesis of 2-Dur

A mixture of **1** (126 mg, 247 μmol) and H_2B-Dur (36 mg, 247 μmol) was stirred in 10 mL benzene for 3 h at room temperature. The reaction mixture turned yellow. Insoluble residues were removed by filtration, volatiles were removed under reduced pressure. The crude yellow product was washed with hexane (3x 5 mL) and dried *in vacuo* to afford compound **2-Dur** as a yellow solid. (125 mg, 190 μmol, 77%). $^{1}H\{^{11}B\}$ NMR (500 MHz, C_6D_6, 297 K): δ = 6.60 (s, 1H, CH_{Dur}), 6.10, 6.03 (two br s, 2H each, NCH_{IiPr}), 5.72 (br, 2H, CH_{iPr}), 4.80 (m, 2H, CH_{Cp}), 4.73 (br, 2H, CH_{iPr}), 4.62, 4.56, 4.38 (three m, 2H each, CH_{Cp}), 3.04 (s, 2H, B*H*), 2.17 (s, 6H, $CH_{3\text{-Dur}}$), 2.12 (s, 6H, CH_{3}-$_{Dur}$), 1.07, 1.02, 0.76, 0.42 (four br, 6H each, $CH_{3\text{-IiPr}}$) ppm. $^{13}C\{^{1}H\}$ NMR (126 MHz, C_6D_6, 297 K): δ = 174.4 (br, $C_{carbene}$), 160.4 ($C_{q\text{-Dur}}$), 131.8 ($C_{q\text{-Dur}}$), 128.8 ($C_{q\text{-Dur}}$), 126.5 ($CH_{\text{-Dur}}$), 114.0 (NCH_{IiPr}), 113.8 (NCH_{IiPr}), 90.6 (br, $C_{q\text{-Cp}}$), 78.6, 78.0, 69.2, 68.8 (CH_{Cp}) 49.0, 48.6 (CH_{iPr}), 25.0, 23.6, 21.7, 20.8 ($CH_{3\text{-}iPr}$), 20.2, 19.2 ($CH_{3\text{-Dur}}$) ppm. ^{11}B NMR (160 MHz, C_6D_6, 297 K): δ = 107.6 (sp^2-*B*), –14.7 (sp^3-*B*) ppm. Solid-state IR: υ(B*H*) = 2188, 2163 cm^{-1}. Elemental analysis (%) for $C_{38}H_{55}B_3FeN_4$ (M_w = 656.2): calcd. C 69.59, H 8.45, N 8.54; found: C 70.65, H 8.45, N 8.56.

Synthesis of 2'-Mes

10 mg of **2-Mes** dissolved in C_6D_6 in a J.-Young-style NMR tube were irradiated at 480 nm for 18 h. Evaporation of the solvent in the glovebox enabled the isolation of a few crystals of **2'-Mes**. NMR data obtained from the mixture of **2-Mes** and **2'-Mes** obtained after 18 h irradiation. $^{1}H\{^{11}B\}$ NMR (400 MHz, C_6D_6, 297 K): δ = 6.78, 6.59 (two s, 1H each, CH_{Mes}), 6.08 (s, 4H, NCH_{IiPr}), 5.37 (sept, ^3J = 6.7 Hz, 4H, CH_{iPr}), 4.67 (s, 2H, CH_{Cp}), 4.50 (s, 4H, CH_{Cp}), 4.27 (s, 2H, CH_{Cp}), 2.58 ($CH_{3\text{-Mes}}$), 2.58 (s, 2H, B*H*), 2.22, 2.16 (two s, 3H each, $CH_{3\text{-Mes}}$), 0.91, 0.87 (two d, ^3J = 6.7 Hz, 12H each, $CH_{3\text{-}iPr}$). ^{11}B NMR (128 MHz, C_6D_6, 297 K): δ = 78.9, –11.3 ppm.

Synthesis of 2'-Dur

10 mg of **2-Dur** dissolved in C_6D_6 in a J.-Young-style NMR tube were irradiated at 480 nm for 24 h. Evaporation of the solvent in the glovebox enabled the isolation of a few crystals of **2'-Dur**. NMR data were obtained from the mixture of **2-Dur** and **2'-Dur** obtained after 24 h irradiation. $^{1}H\{^{11}B\}$ NMR (400 MHz, C_6D_6, 297 K): δ = 6.61 (s, 2H, CH_{Mes}), 6.06 (s, 4H, NCH_{IiPr}), 5.36 (sept, ^3J = 6.8 Hz, 4H, CH_{iPr}), 4.66 (s, 2H, CH_{Cp}), 4.51 (s, 4H, CH_{Cp}), 4.33 (s,

2H, CH_{Cp}), 2.65 (s, 2H, BH), 2.49, 2.21, 2.11, 2.10 (four s, 3H each, C$H_{3\text{-Mes}}$), 0.90, 0.86 (two d, ^3J = 6.7 Hz, 12H each, C$H_{3\text{-}i\text{Pr}}$). ^{11}B NMR (128 MHz, C_6D_6, 297 K): δ = 88.8, –12.5 ppm.

Synthesis of 3-Mes

A solution of **2-Mes** (63 mg, 98 μmol) in 1 mL of dichloromethane was treated with a solution of MeOTf (20 mg, 122 μmol) in 1 mL of dichloromethane. The mixture was stirred for 1 h at room temperature and turned deep red during this time. Volatiles were removed under reduced pressure, the crude red product was recrystallized from a dichloromethane/benzene solution (9:1) by slow evaporation of the solvent to afford compound **3-Mes** as a red crystalline solid (63 mg, 76 μmol, 81%). ^{1}H{^{11}B} NMR (500 MHz, C_6D_6, 233 K): δ = 7.37 (d, ^3J = 1.7 Hz, 1H, C$H_{\text{I}i\text{Pr}}$), 7.24 (d, ^3J = 1.7 Hz, 1H, C$H_{\text{I}i\text{Pr}}$), 6.98 (d, ^3J = 1.7 Hz, 1H, C$H_{\text{I}i\text{Pr}}$), 6.85 (d, ^3J = 1.7 Hz, 1H, C$H_{\text{I}i\text{Pr}}$), 6.70 (s, 1H, CH_{Mes}), 6.55 (s, 1H, CH_{Mes}), 5.48 (sept, ^3J = 6.7 Hz, 1H, C$H_{i\text{Pr}}$), 5.05 (s, 1H, CH_{Cp}), 4.92 (m, 2H, CH_{Cp} + C$H_{i\text{Pr}}$), 4.45 (m, 1H, CH_{Cp}), 4.42 (m, 2H, CH_{Cp} + C$H_{i\text{Pr}}$), 4.27 (s, 1H, CH_{Cp}), 4.19 (m, 1H, CH_{Cp}), 4.17 (m, 2H, CH_{Cp} + C$H_{i\text{Pr}}$), 3.43 (s, 1H, CH_{Cp}), 2.31 (s, 3H, C$H_{3\text{-Mes}}$), 2.07 (s, 3H, C$H_{3\text{-Mes}}$), 1.78 (s, 3H, C$H_{3\text{-Mes}}$), 1.70 (d, ^3J = 6.6 Hz, 3H, C$H_{3\text{-I}i\text{Pr}}$), 1.58 (d, ^3J = 6.7 Hz, 3H, C$H_{3\text{-I}i\text{Pr}}$), 1.40 (d, ^3J = 6.8 Hz, 3H, C$H_{3\text{-I}i\text{Pr}}$), 1.25 (d, ^3J = 6.7 Hz, 3H, C$H_{3\text{-I}i\text{Pr}}$), 1.15 (d, ^3J = 6.7 Hz, 3H, C$H_{3\text{-I}i\text{Pr}}$), 0.88 (m, 6H, C$H_{3\text{-I}i\text{Pr}}$), 0.63 (s, 1H, BHB), 0.44 (d, ^3J = 6.5 Hz, 3H, C$H_{3\text{-I}i\text{Pr}}$) ppm. ^{13}C{^{1}H} NMR (126 MHz, C_6D_6, 233 K): δ = 157.2 (br, C_{carbene}), 155.9 (br, C_{carbene}), 141.8, 139.4, 137.9, 135.1 ($C_{\text{q-Mes}}$), 128.3 (C_6H_6), 127.2 (CH$_{\text{-Mes}}$), 127.0 (CH$_{\text{-Mes}}$), 120.6 (q, $^1J_{\text{C-F}}$ = 320 Hz, CF$_3$), 119.3 (CH$_{\text{I}i\text{Pr}}$), 118.7 (CH$_{\text{I}i\text{Pr}}$), 116.0 (CH$_{\text{I}i\text{Pr}}$), 115.9 (CH$_{\text{I}i\text{Pr}}$), 85.0 (br, $C_{q\text{-Cp}}$), 83.3, 82.5, 81.0 (CH$_{\text{Cp}}$), 80.7 (br, $C_{q\text{-Cp}}$), 76.4, 74.5, 73.5, 70.3, 69.2 (CH$_{\text{Cp}}$), 51.24, 51.21, 50.5, 50.4 (CH$_{i\text{Pr}}$), 25.9 (CH$_{3\text{-Mes}}$), 25.1, 24.4, 23.9, 22.9 (CH$_{3\text{-}i\text{Pr}}$), 22.8 (CH$_{3\text{-Mes}}$), 22.7, 22.5, 21.5 (CH$_{3\text{-}i\text{Pr}}$), 20.6 (CH$_{3\text{-Mes}}$), 20.5 (CH$_{3\text{-}i\text{Pr}}$) ppm. ^{11}B NMR (160 MHz, C_6D_6, 297 K): δ = 80.8 (sp^2-$B3$), 46.6 (Mes$B2$), 22.2 (H$B1$) ppm. ^{19}F NMR (470 MHz, CD_2Cl_2, 233K) δ = –79.19 (s, CF_3) ppm. Solid-state IR: υ(BH) = 1568 cm^{-1}. Elemental analysis (%) for $C_{38}H_{52}B_3FeN_4O_3SF_3$ (M_w = 790.2): calcd. C 57.76, H 6.63, N 7.09, S 4.06; found: C 58.80.46, H 6.75, N 6.74, S 3.96.

Synthesis of 3-Dur

A solution of **2-Dur** (63 mg, 98 μmol) in 1 mL of dichloromethane was treated with a solution of MeOTf (20 mg, 122 μmol) in 1 mL of dichloromethane. The mixture was stirred for 1 h at room temperature and turned deep red during this time. Volatiles were removed under reduced pressure, and the crude red product was recrystallized from a dichloromethane/benzene solution (9:1) by slow evaporation of the solvent to afford compound **3-Dur** as a red crystalline

solid. (47 mg, 58 μmol, 85%). ^{1}H{^{11}B} NMR (500 MHz, CD_2Cl_2, 233 K): δ = 7.38 (m, ^{3}J = 1.4 Hz, 1H, CH_{IiPr}), 7.19 (m, ^{3}J = 1.4 Hz, 1H, CH_{IiPr}), 6.96 (m, ^{3}J = 1.5 Hz, 1H, CH_{IiPr}), 6.80 (m, ^{3}J = 1.5 Hz, 1H, CH_{IiPr}), 6.62 (s, 1H, CH_{Dur}), 5.45 (m, sept, ^{3}J = 6.7 Hz, 1H, CH_{iPr}), 5.18 (s, 1H, CH_{Cp}), 4.94 (m, 2H, CH_{Cp} + CH_{iPr}), 4.46 (m, 2H, CH_{Cp}), 4.30 (s, 1H, CH_{Cp}), 4.16-4.23 (m, 4H, CH_{Cp} + CH_{iPr}), 3.47 (s, 1H, CH_{Cp}), 2.32 (s, 3H, C$H_{3\text{-Dur}}$), 2.09 (s, 3H, C$H_{3\text{-Dur}}$), 1.91 (s, 3H, C$H_{3\text{-Dur}}$), 1.76 (s, 3H, C$H_{3\text{-Dur}}$), 1.73 (d, ^{3}J = 6.5 Hz, 3H, C$H_{3\text{-IiPr}}$), 1.63 (d, ^{3}J = 6.7 Hz, 3H, C$H_{3\text{-IiPr}}$), 1.42 (d, ^{3}J = 6.7 Hz, 3H, C$H_{3\text{-IiPr}}$), 1.24 (d, ^{3}J = 6.6 Hz, 3H, C$H_{3\text{-IiPr}}$), 1.09 (d, ^{3}J = 6.8 Hz, 3H, C$H_{3\text{-IiPr}}$), 0.89 (d, ^{3}J = 6.5 Hz, 3H, C$H_{3\text{-IiPr}}$), 0.81 (s, br, 1H, B*H*B), 0.71 (d, ^{3}J = 6.6 Hz, 3H, C$H_{3\text{-IiPr}}$), 0.36 (d, ^{3}J = 6.7 Hz, 3H, C$H_{3\text{-IiPr}}$) ppm. ^{13}C{^{1}H} NMR (126 MHz, CD_2Cl_2, 233 K): δ = 157.0 (br, $C_{carbene}$), 156.1 (br, $C_{carbene}$), 145.3, 134.3, 133.8, 132.7 ($C_{q\text{-Dur}}$), 129.0 (CH$_{\text{-Dur}}$), 128.3 (C_6H_6), 120.5 (q, $^{1}J_{C\text{-}F}$ = 321 Hz, CF$_3$), 118.9, 118.4, 115.9, 115.8 (CH$_{IiPr}$), 84.7 (br, $C_{q\text{-Cp}}$), 83.3, 82.2, 81.3 (CH$_{Cp}$), 79.6 (br, $C_{q\text{-Cp}}$), 76.1, 74.6, 73.5, 70.3, 68.9 (CH$_{Cp}$), 51.4, 51.2, 50.4, 50.3 (CH$_{iPr}$), 25.0, 24.4, 24.0, 22.7, 22.0, 21.5 (CH$_{3\text{-}iPr}$), 21.2 , 20.5 (CH$_{3\text{-Dur}}$), 20.4 (CH$_{3\text{-}iPr}$), 20.27, 20.25 (CH$_{3\text{-Dur}}$) ppm. ^{11}B NMR (160 MHz, CD_2Cl_2, 297 K): δ = 78.2 (sp^{2}-*B3*), 45.9 (Dur*B2*), 19.6 (H*B1*) ppm. ^{19}F NMR (470 MHz, CD_2Cl_2, 233K) δ = –79.18 (s, C*F*$_3$) ppm. Solid-state IR: υ(B*H*) = 1563 cm^{-1}. Elemental analysis (%) for $C_{39}H_{54}B_3FeN_4O_3SF_3{\cdot}C_6H_6$ (M_w = 882.3): calcd. C 61.26, H 6.85, N 6.35, S 3.63; found: C 61.46, H 6.73, N 6.59, S 3.53.

<u>**NMR spectra of isolated compounds**</u>

Figure S1. $^{1}H\{^{11}B\}$ NMR spectrum of **2-Pyr** in C_6D_6.

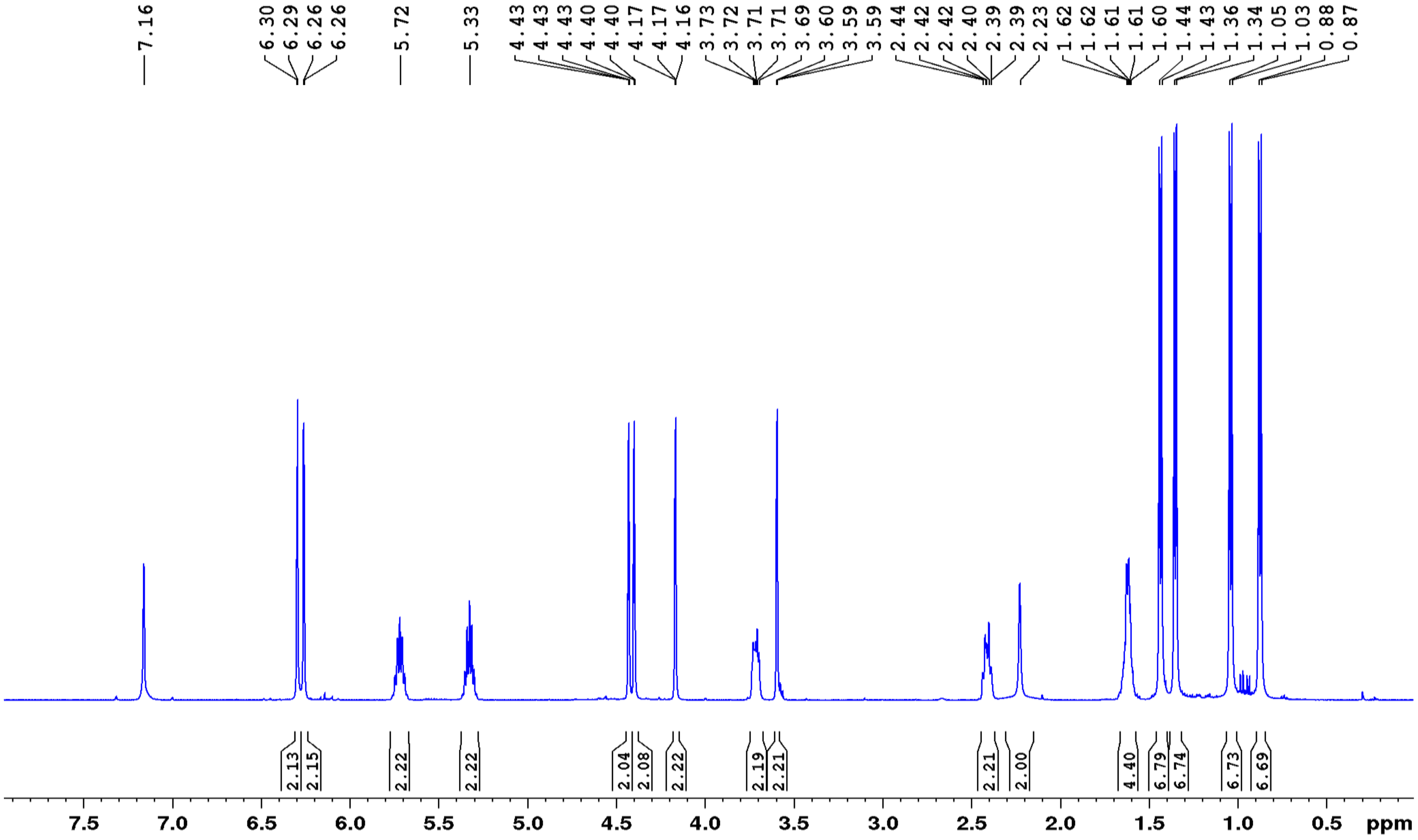

Figure S2. $^{13}C\{^{1}H\}$ NMR spectrum of **2-Pyr** in C_6D_6.

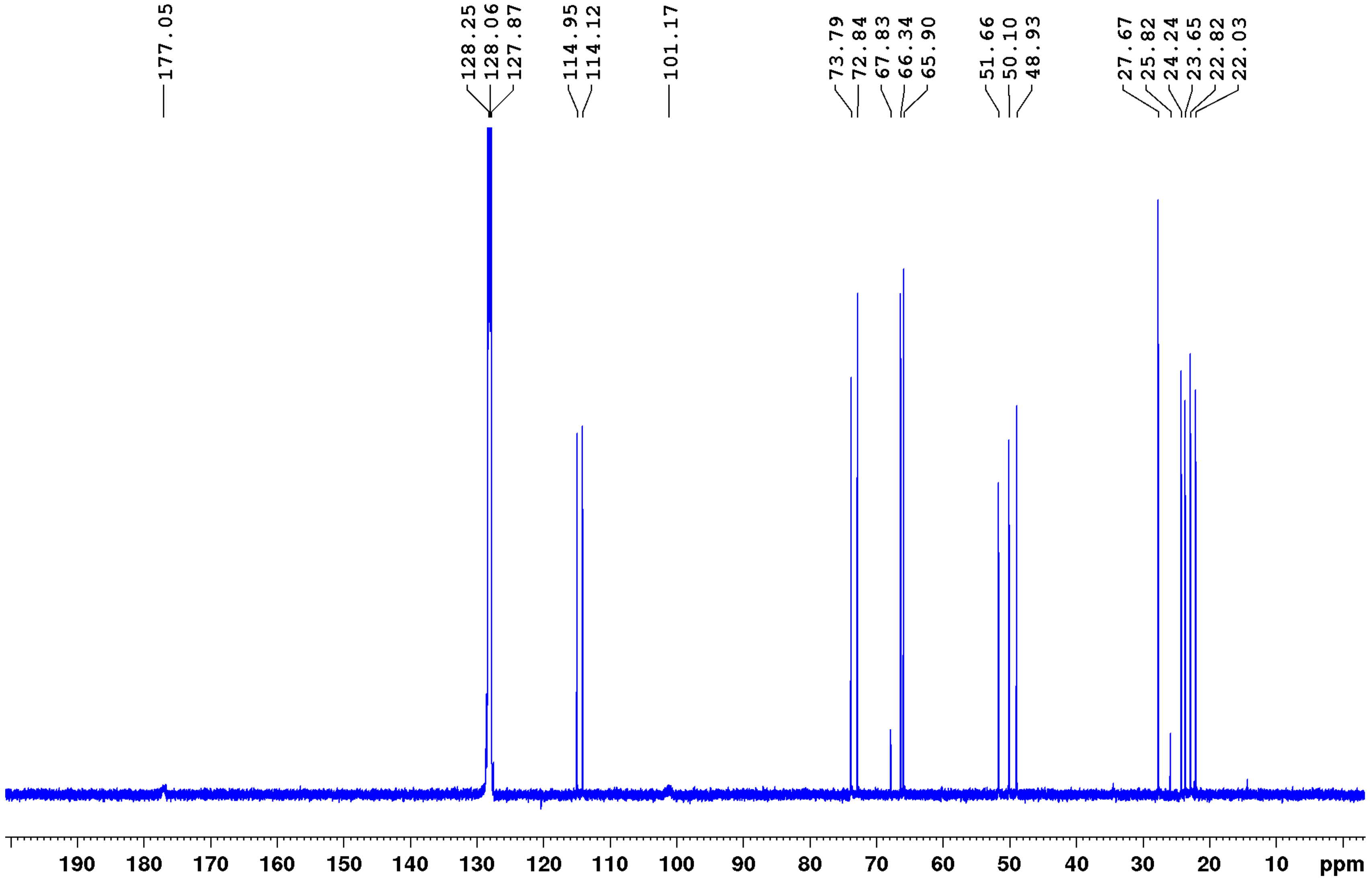

Figure S3. ^{11}B NMR spectrum of **2-Pyr** in C_6D_6.

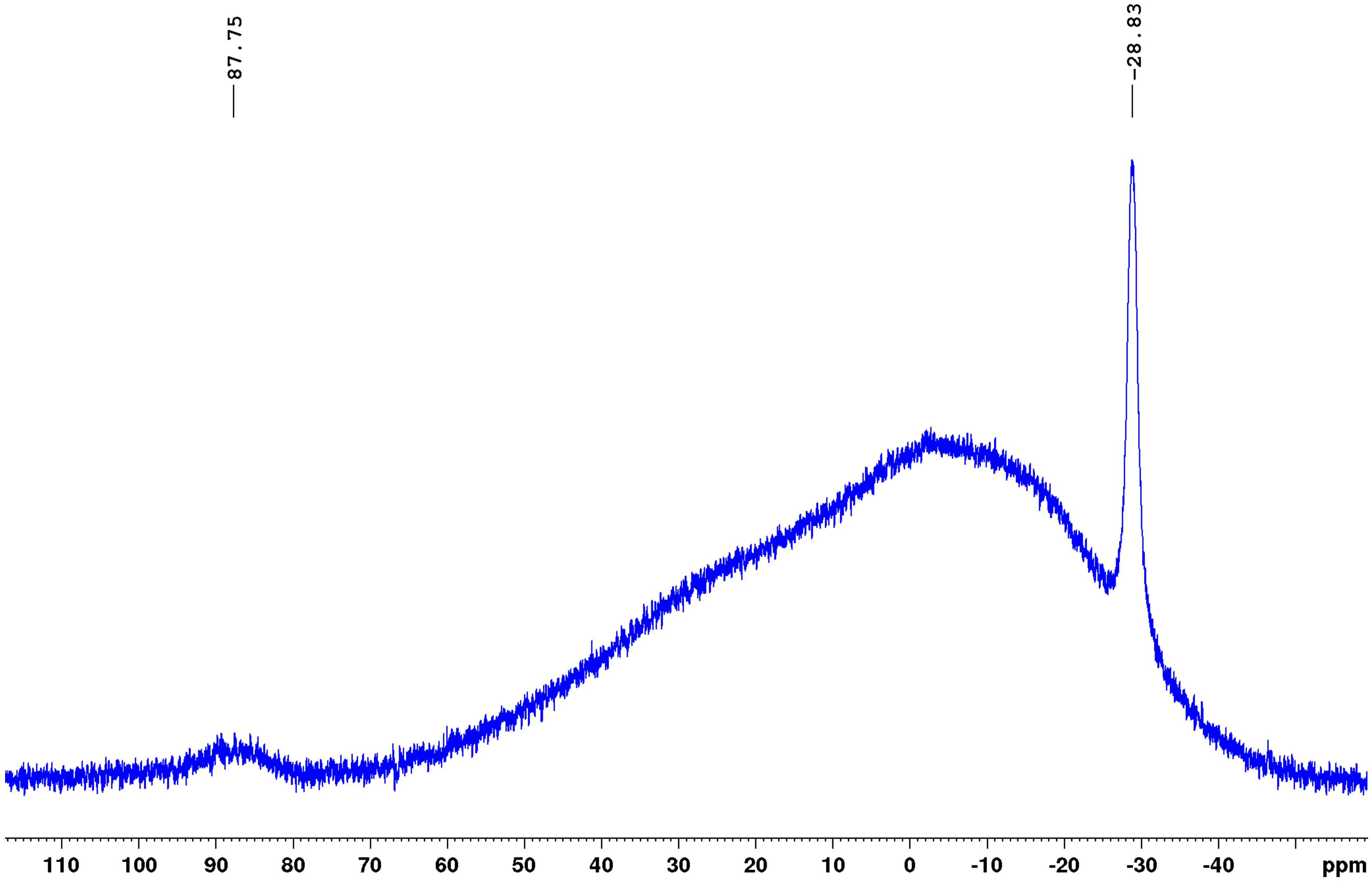

Figure S4. $^{1}H\{^{11}B\}$ NMR spectrum of **2-Mes** in C_6D_6.

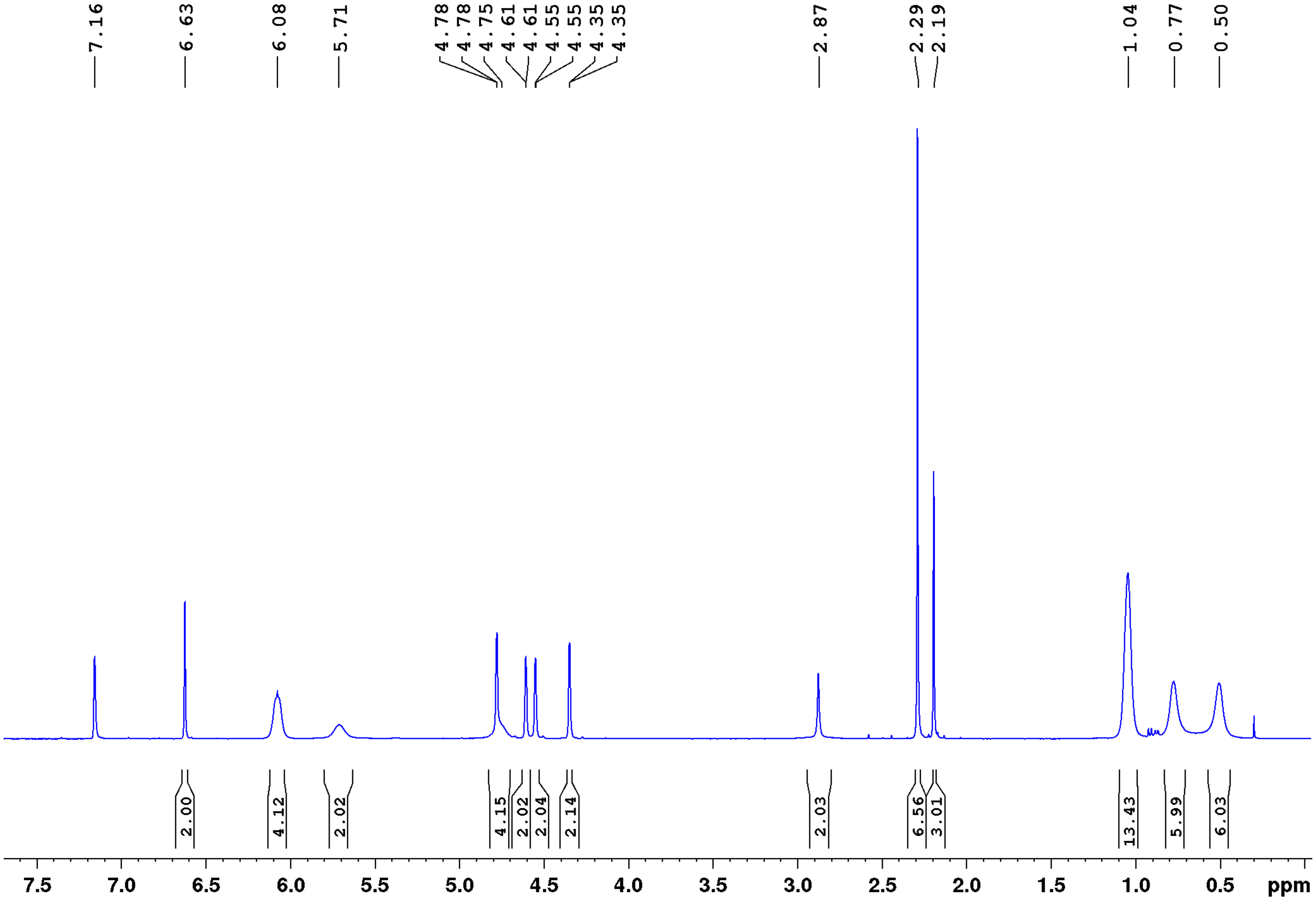

Figure S5. $^{13}C\{^{1}H\}$ NMR spectrum of **2-Mes** in C_6D_6.

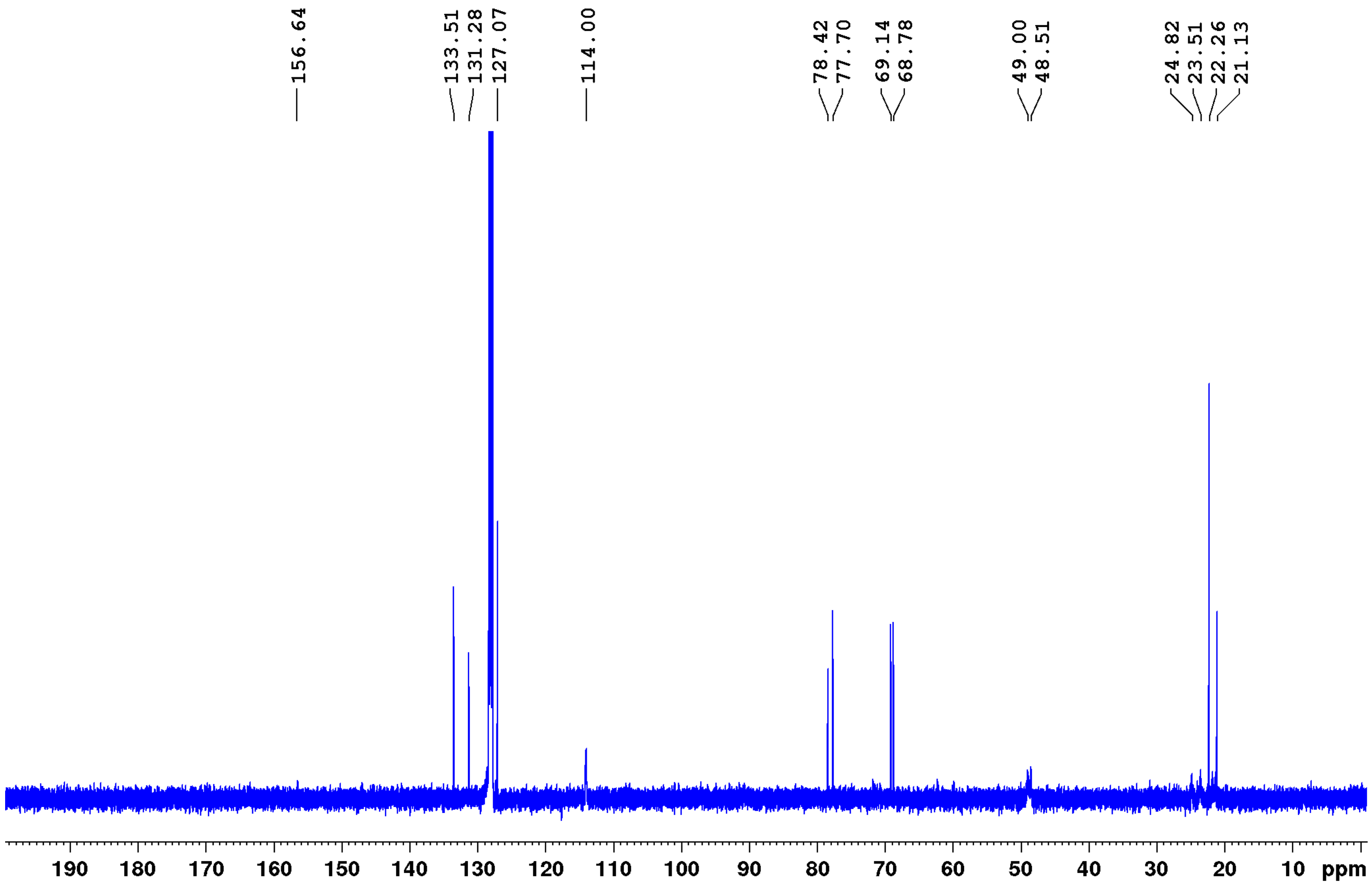

Figure S6. ^{11}B NMR spectrum of **2-Mes** in C_6D_6.

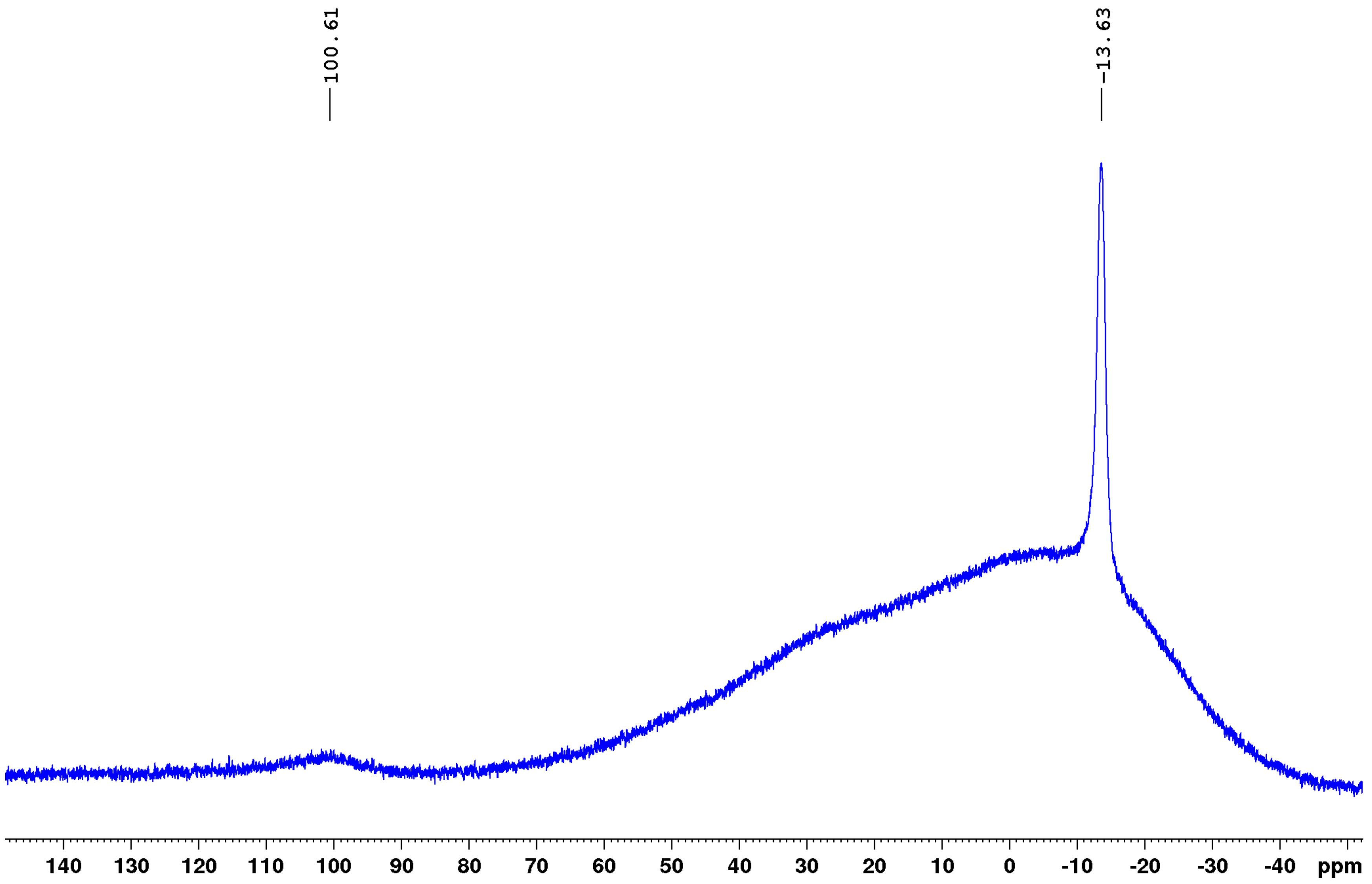

Figure S7. $^{1}H\{^{11}B\}$ NMR spectrum of **2-Dur** in C_6D_6.

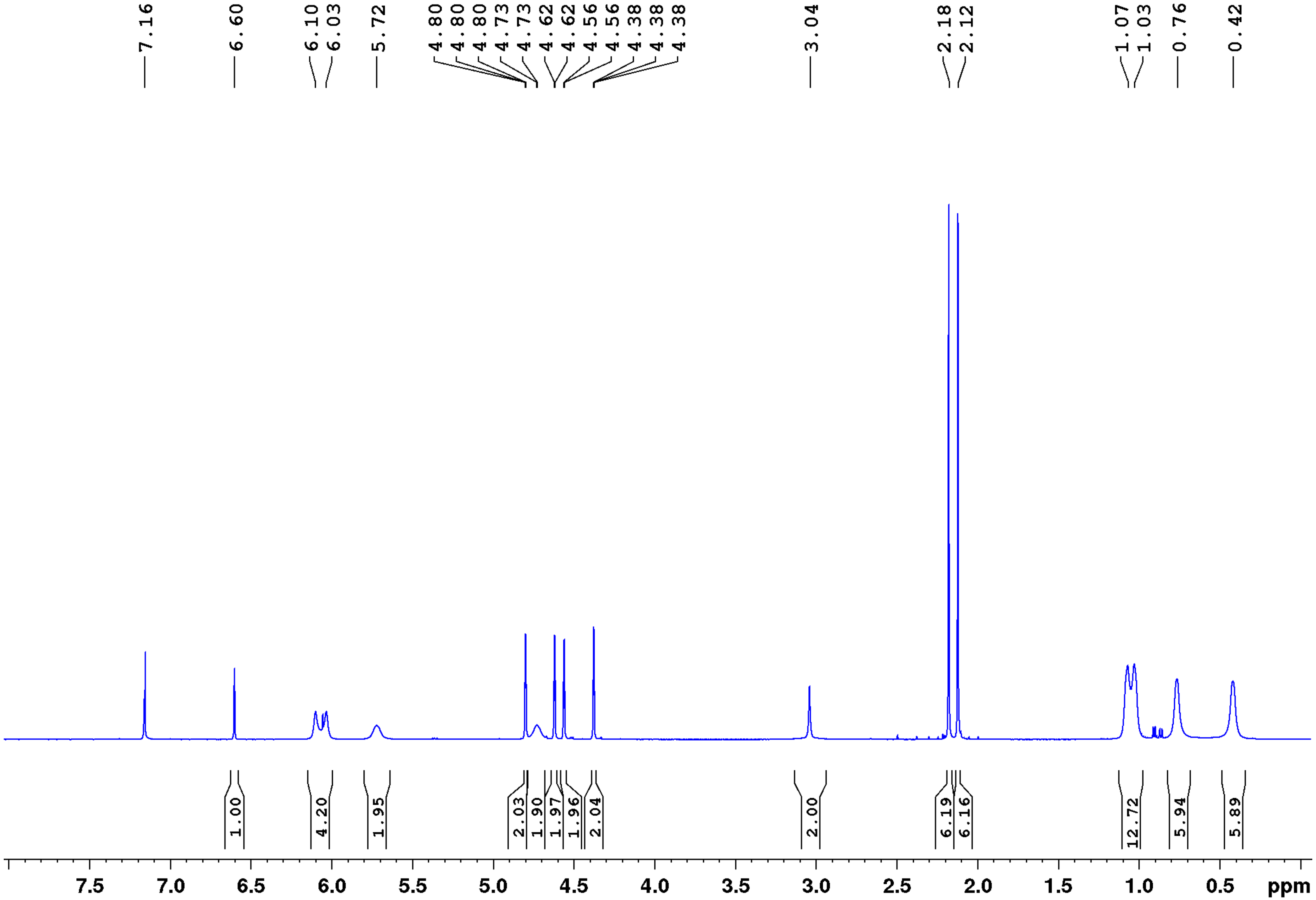

Figure S8. $^{13}C\{^{1}H\}$ NMR spectrum of **2-Dur** in C_6D_6.

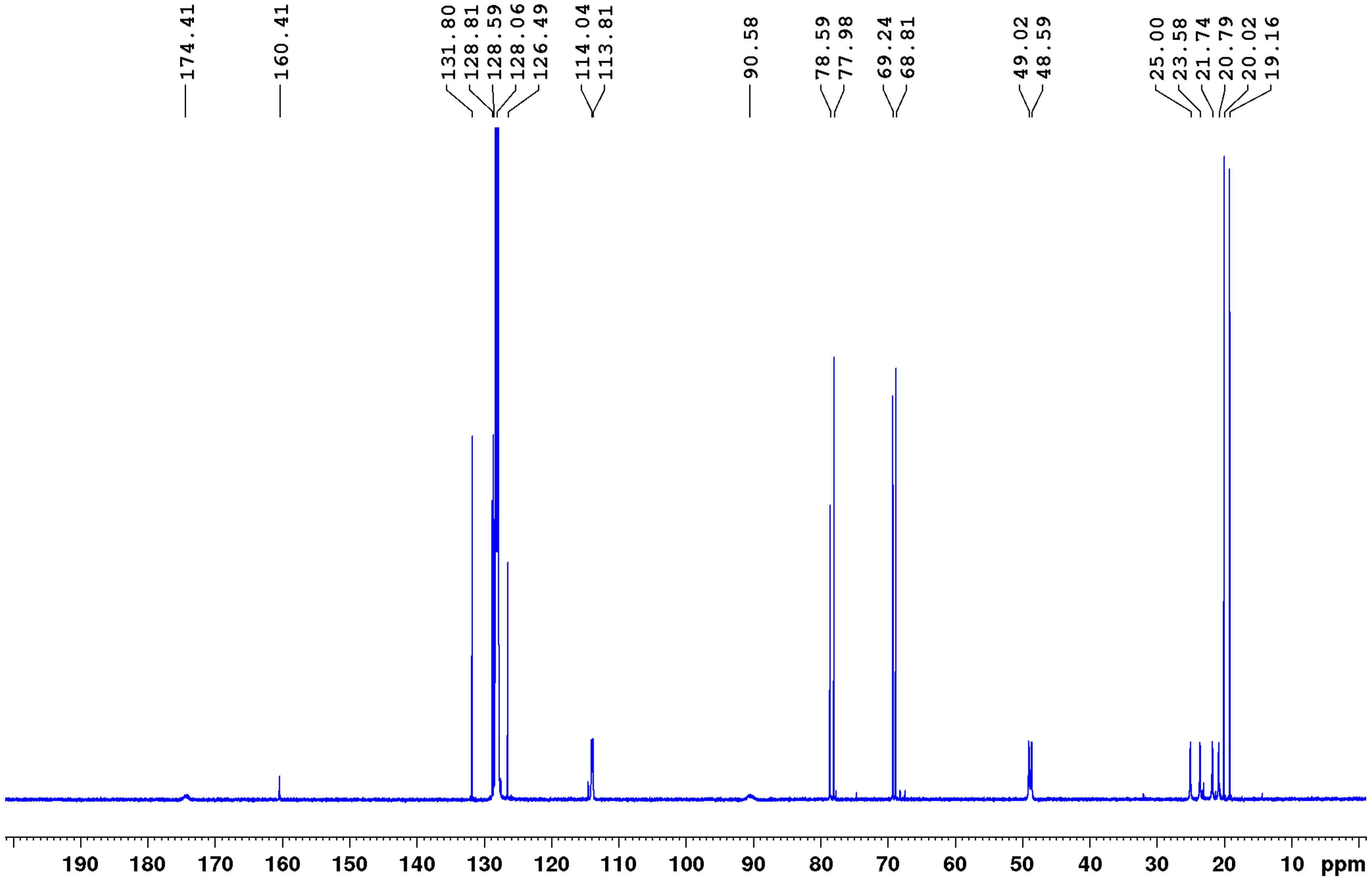

Figure S9. ^{11}B NMR spectrum of **2-Dur** in C_6D_6.

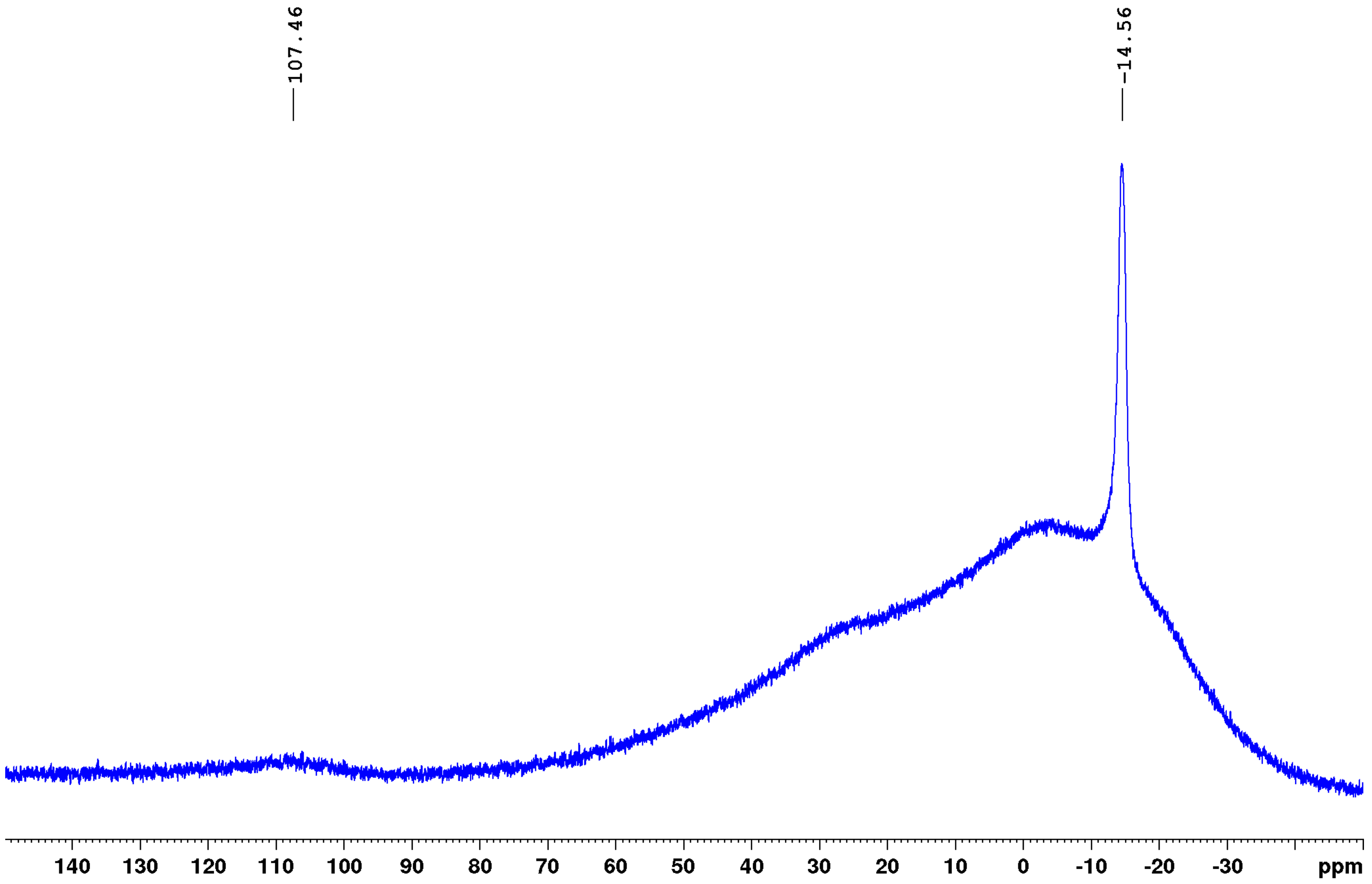

Figure S10. $^{1}H\{^{11}B\}$ NMR spectrum of **3-Mes** in CD_2Cl_2 at –40 °C.

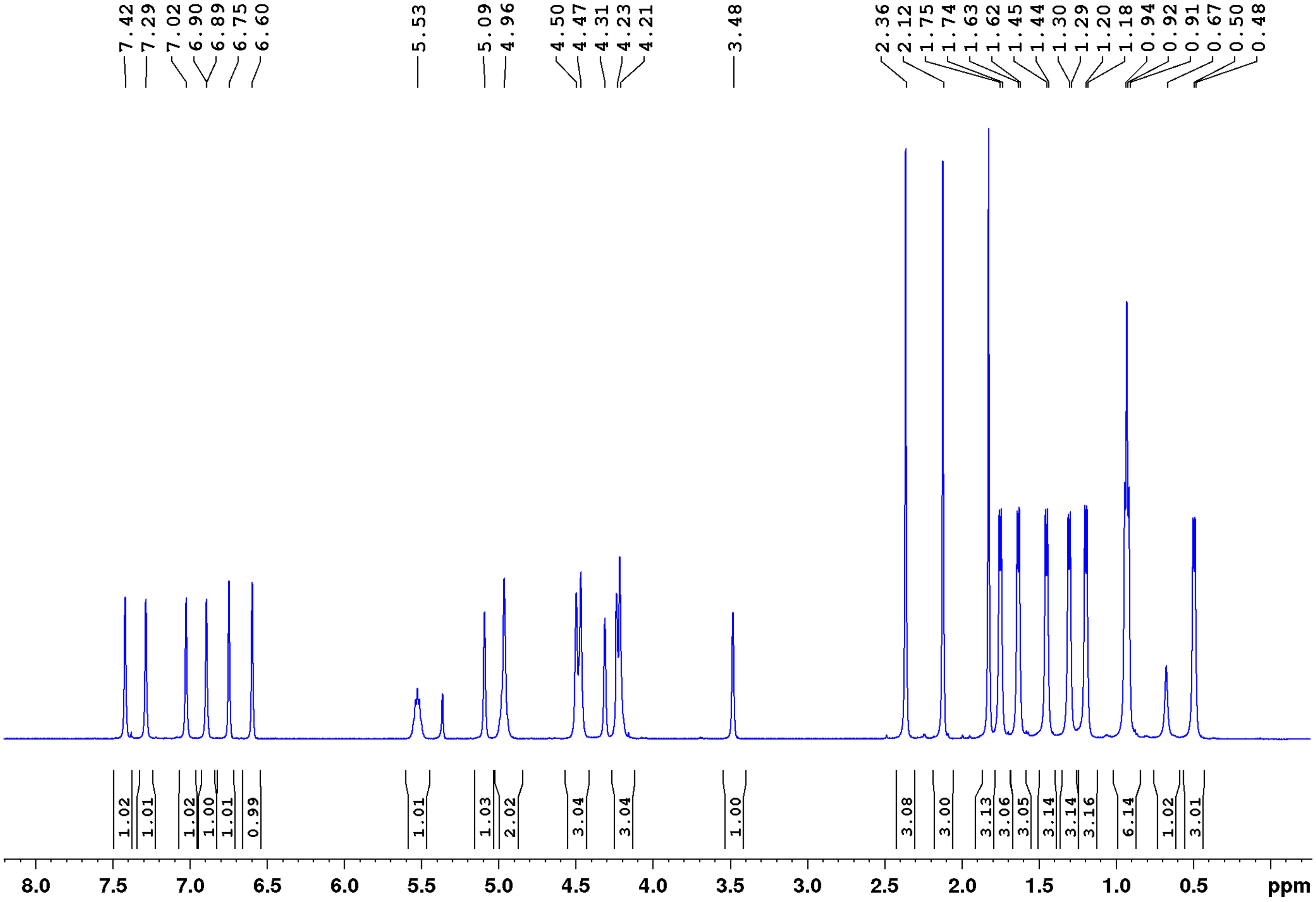

Figure S11. $^{13}C\{^{1}H\}$ NMR spectrum of **3-Mes** in CD_2Cl_2 at –40 °C.

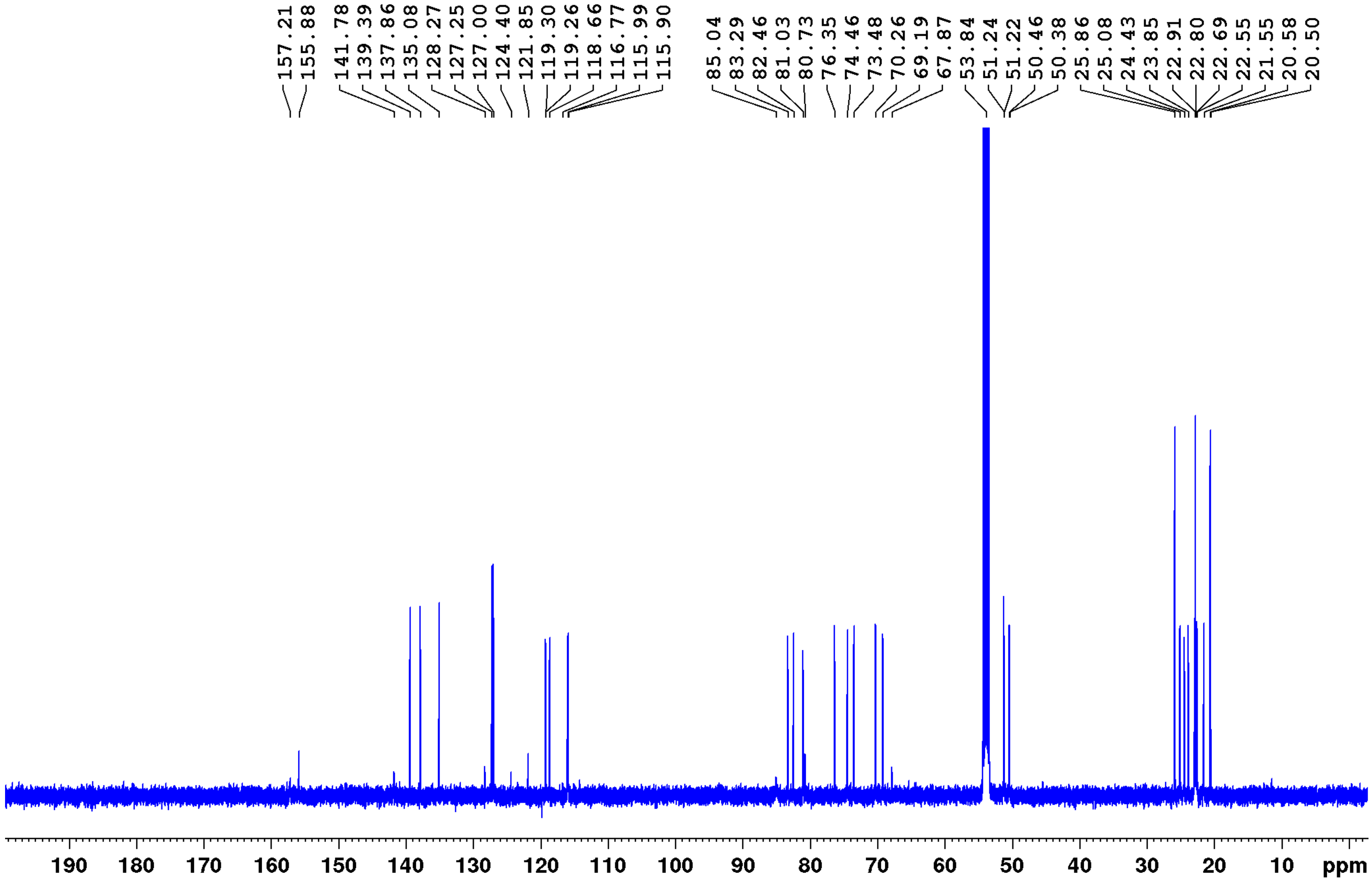

Figure S12. ^{11}B NMR spectrum of **3-Mes** in CD_2Cl_2 at rt.

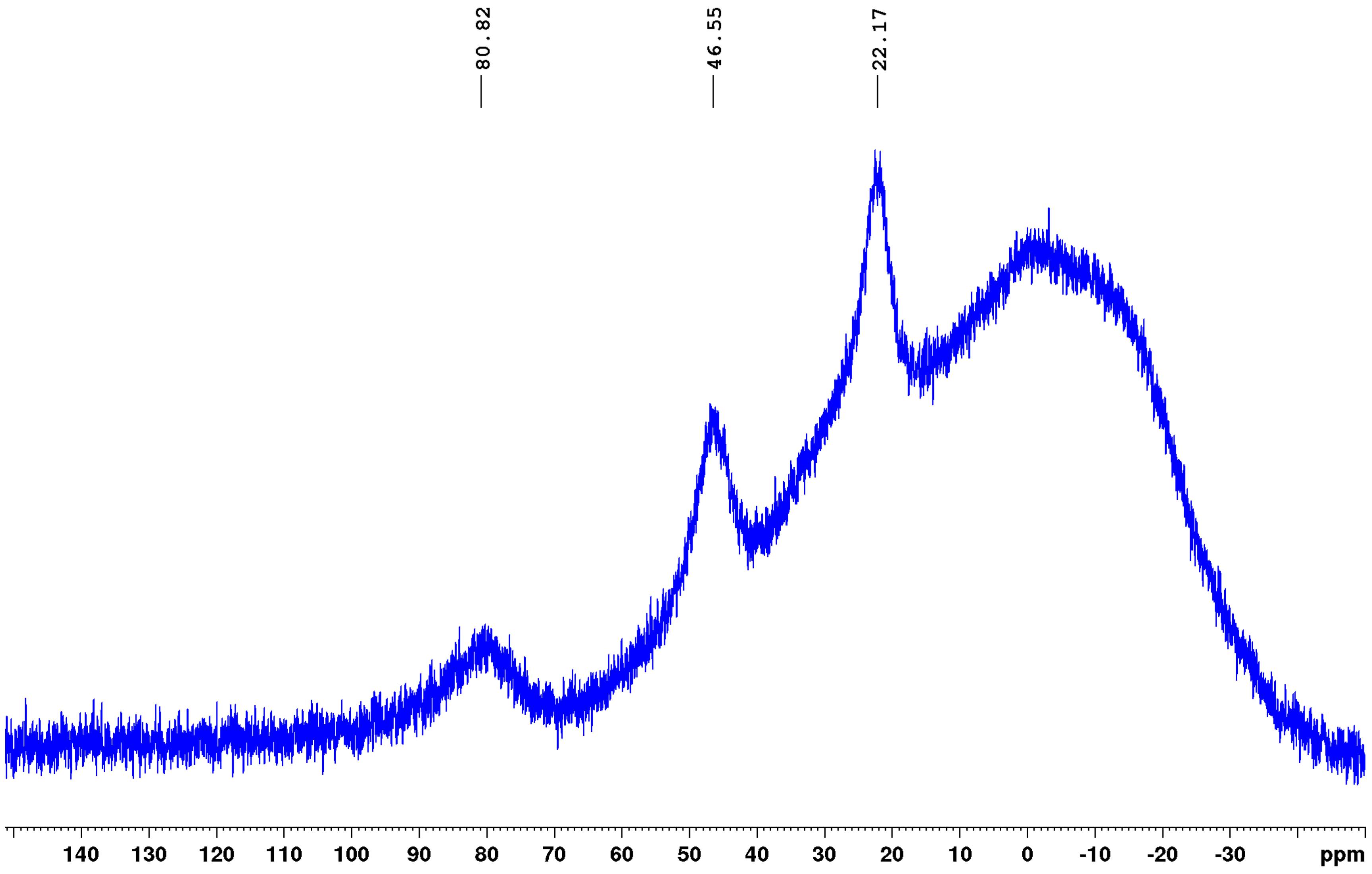

Figure S13. ^{19}F NMR spectrum of **3-Mes** in CD_2Cl_2 at rt.

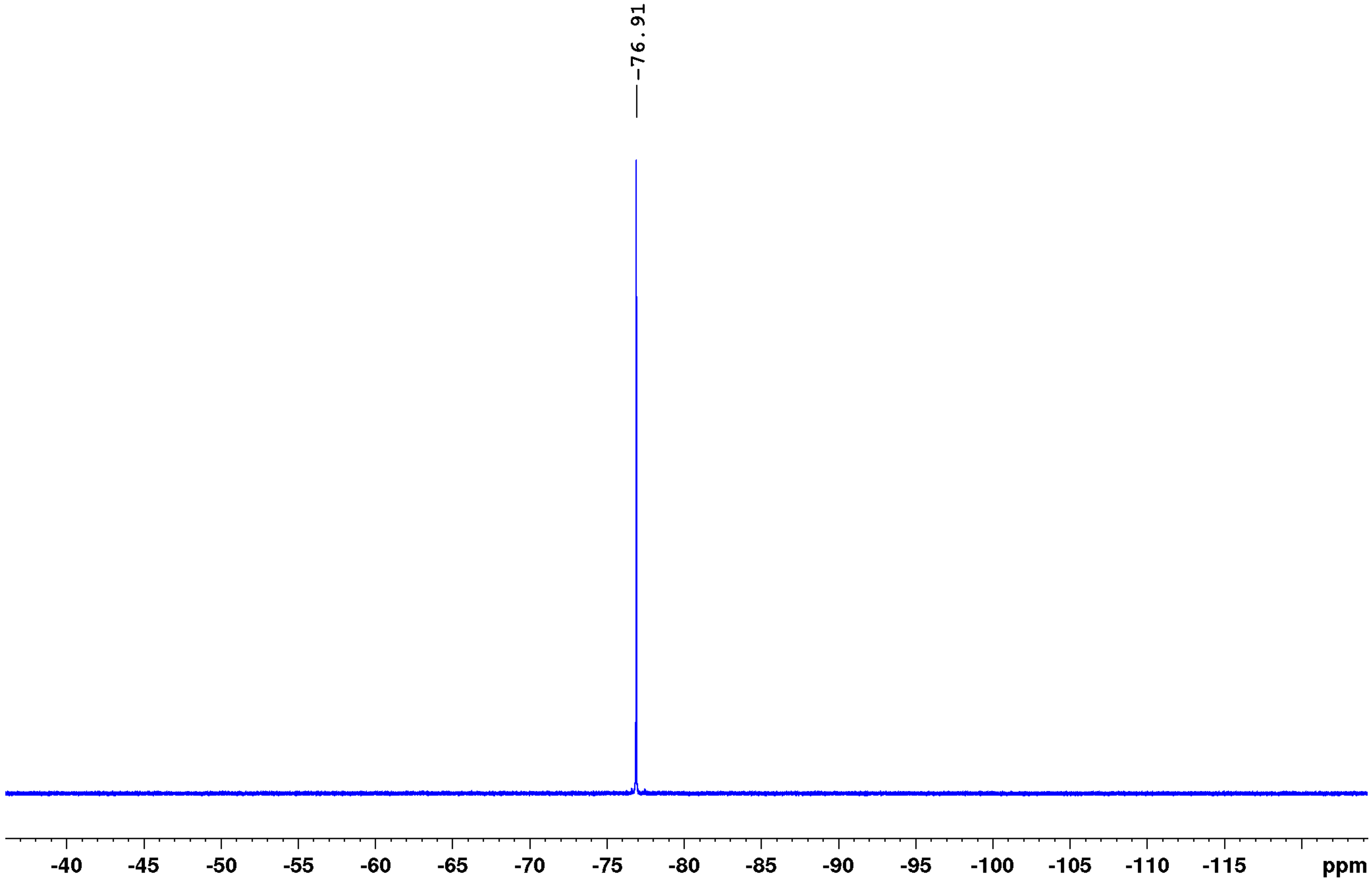

Figure S14. $^{1}H\{^{11}B\}$ NMR spectrum of **3-Dur** in CD_2Cl_2 at –40 °C.

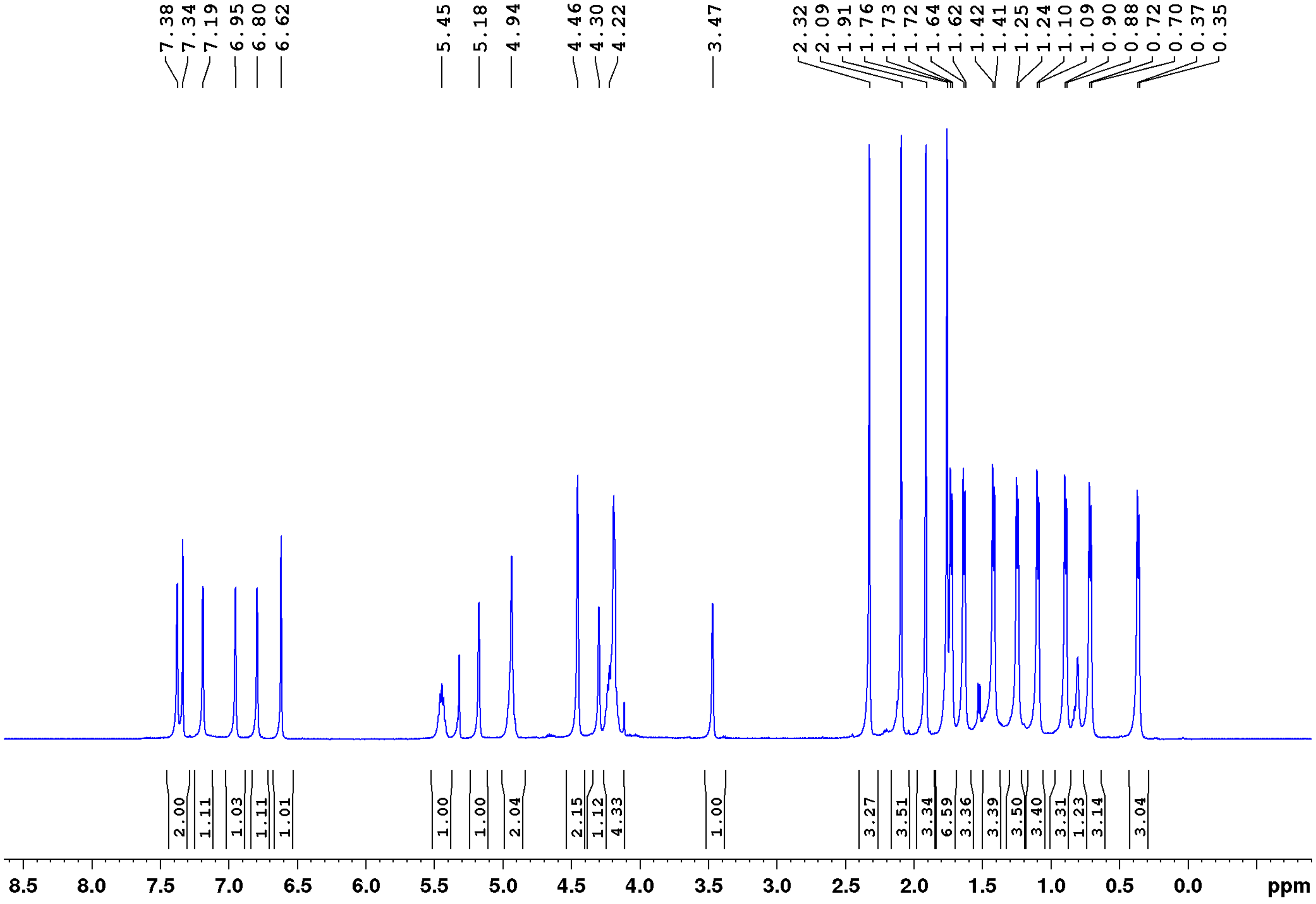

Figure S15. $^{13}C\{^{1}H\}$ NMR spectrum of **3-Dur** in CD_2Cl_2 at –40 °C.

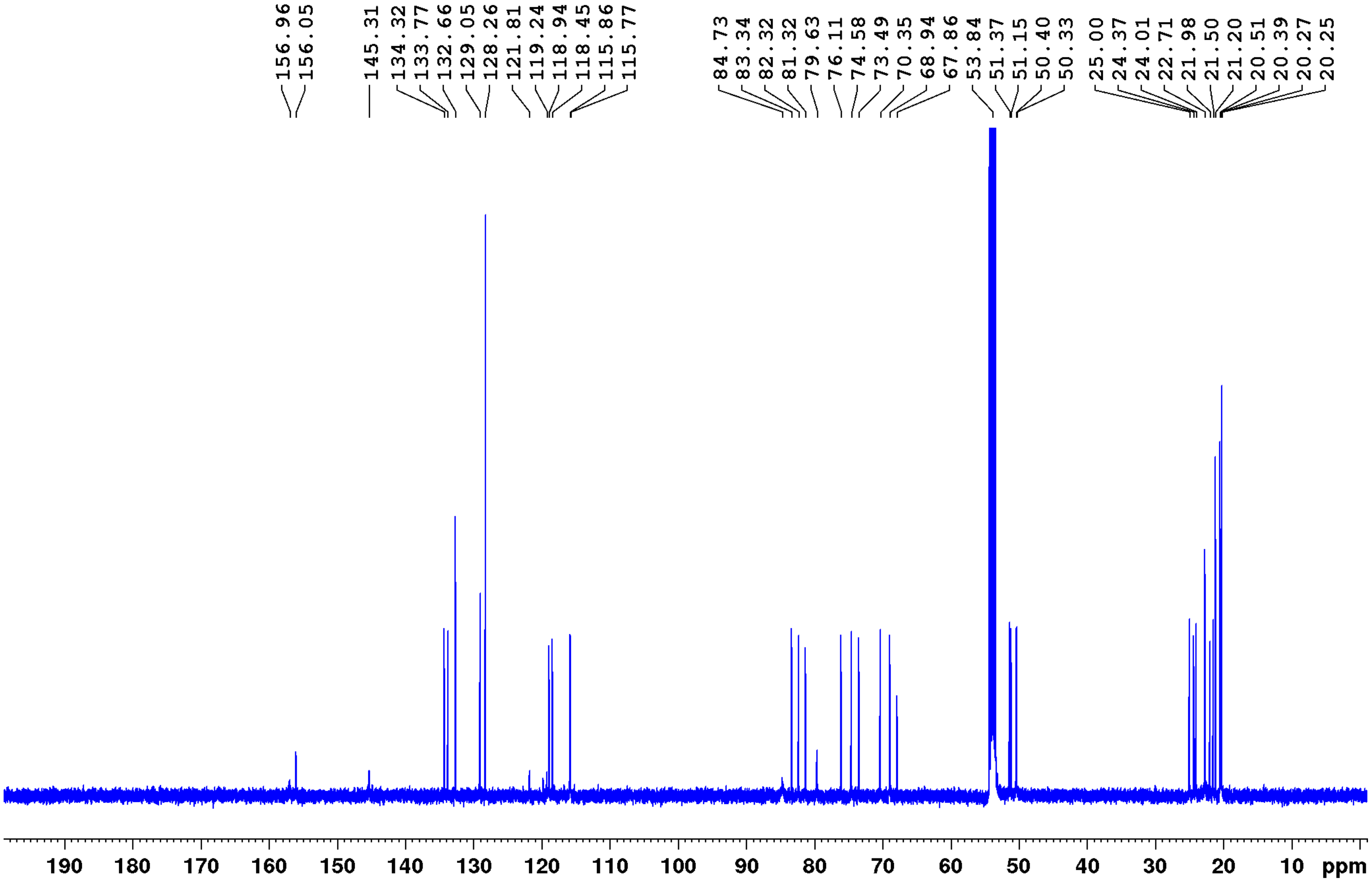

Figure S16. ^{11}B NMR spectrum of **3-Dur** in CD_2Cl_2 at –40 °C.

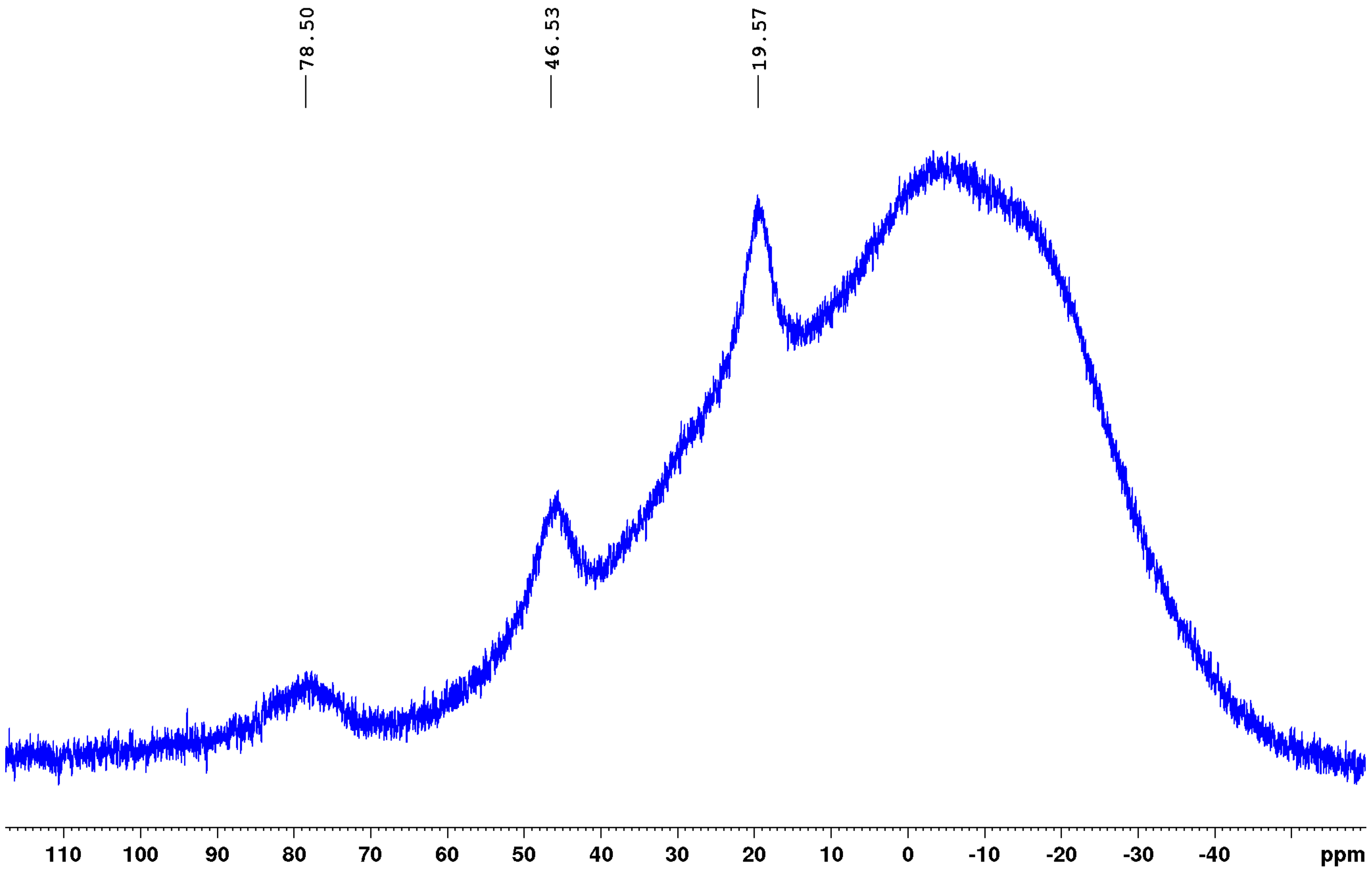

Figure S17. ^{19}F NMR spectrum of **3-Dur** in CD_2Cl_2 at –40 °C.

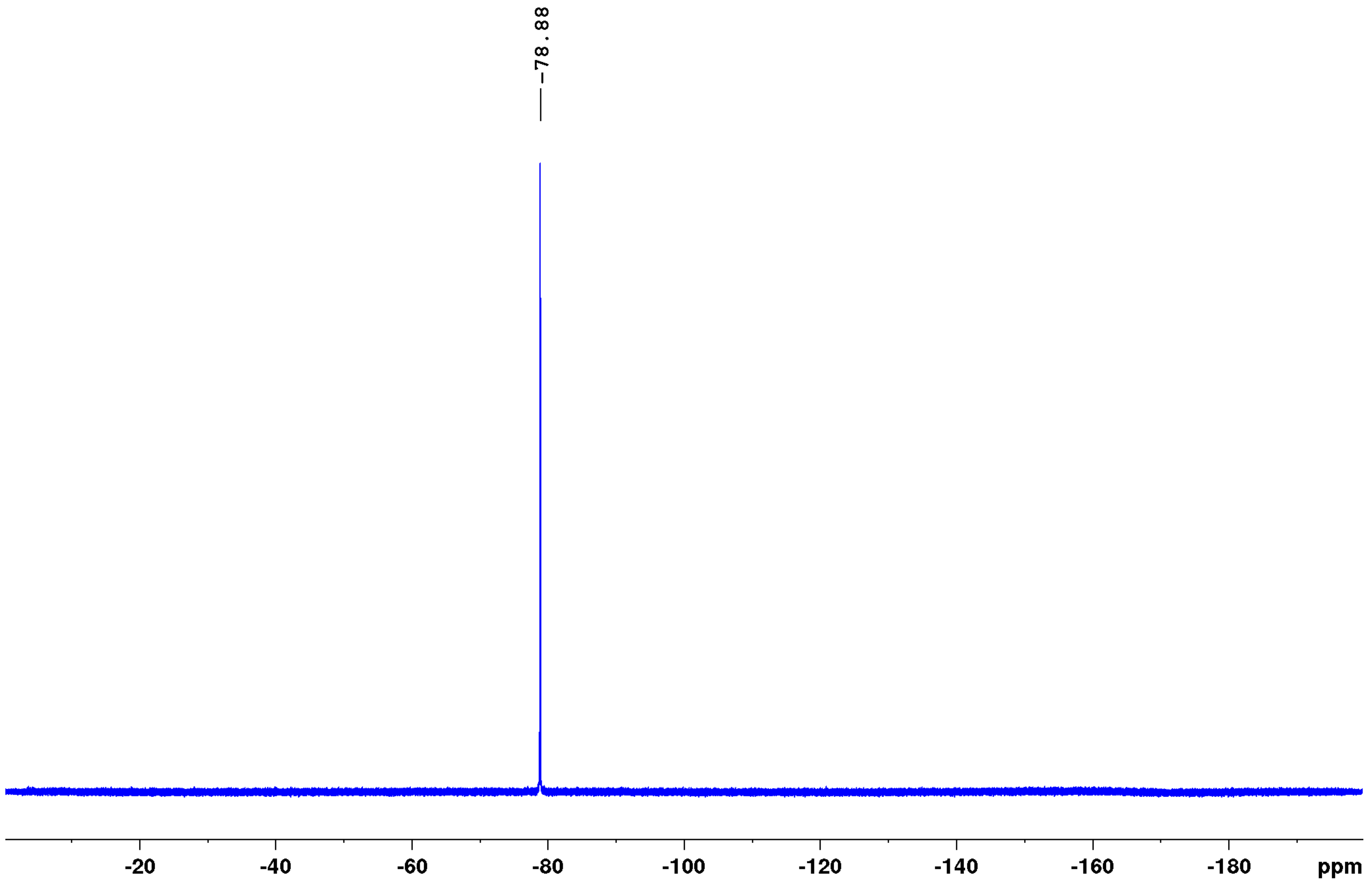

NMR spectra of irradiated samples

Figure S18. Stack-plot of ^{11}B NMR spectra of irradiated samples of **2-Mes** (■) in C_6D_6 (**2'-Mes** ♦).

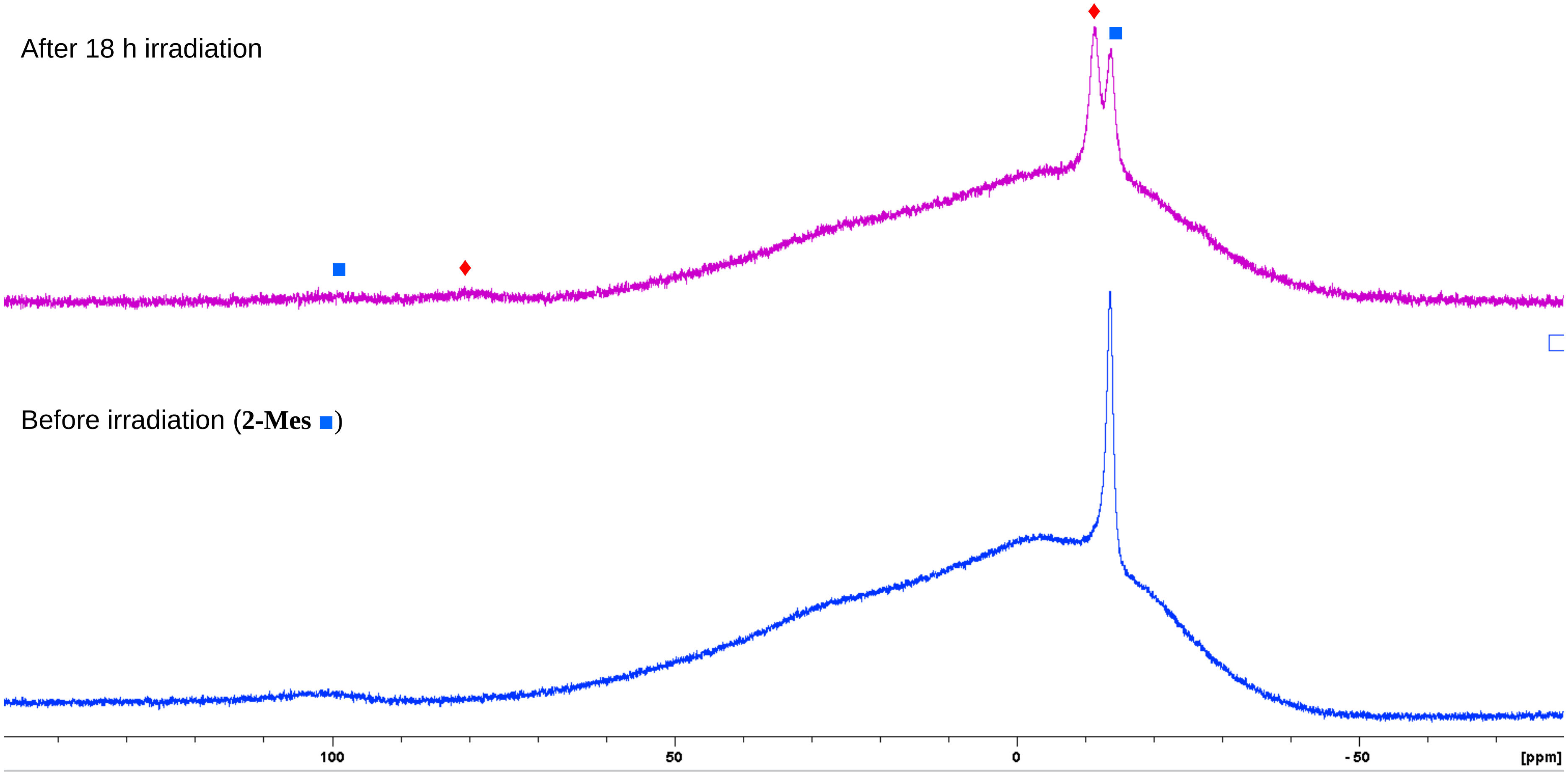

Figure S19. Stack-plot of $^{1}H\{^{11}B\}$ NMR spectra of irradiated samples of **2-Mes** (■) in C_6D_6 (**2'-Mes** ♦).

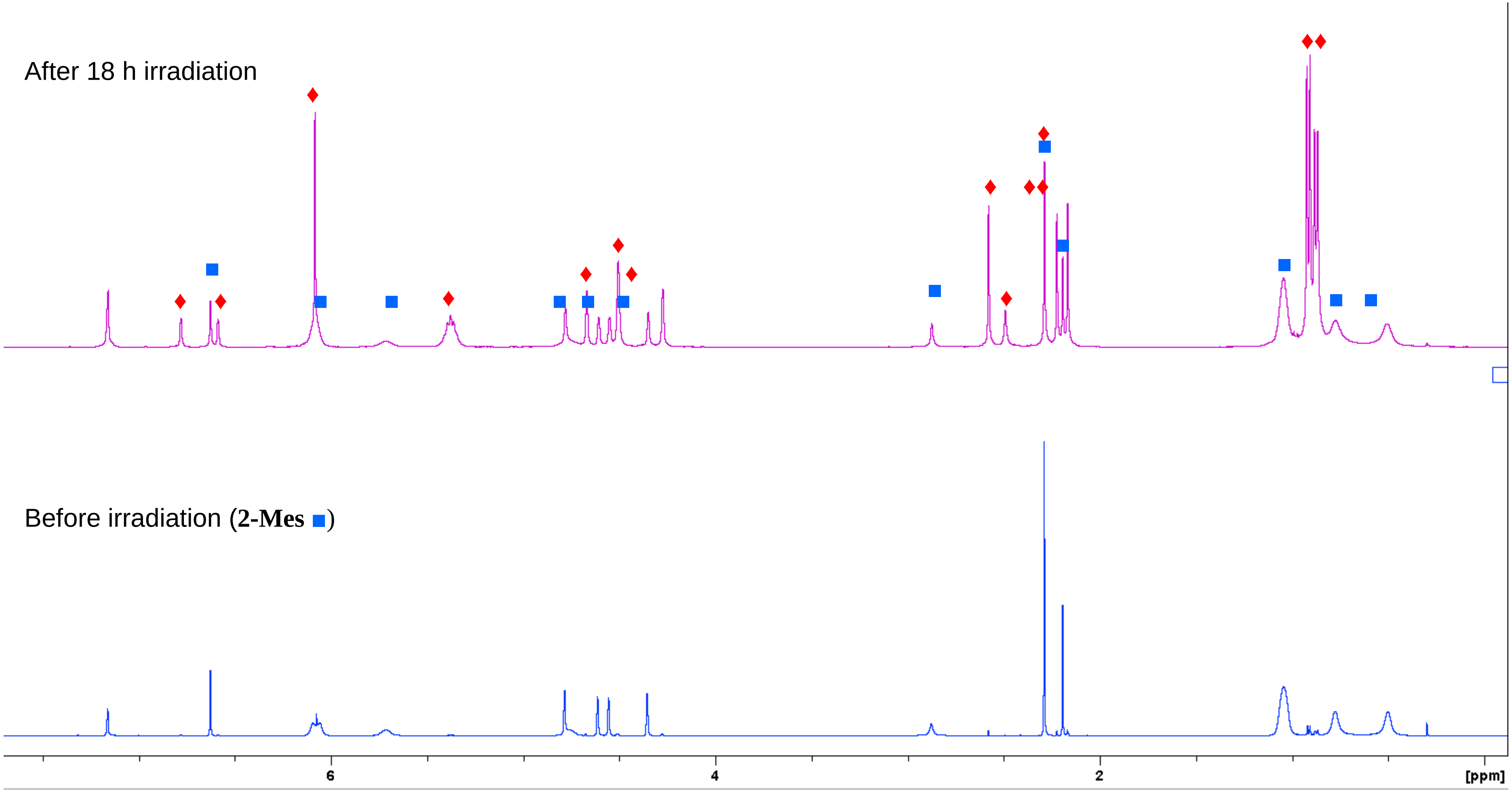

Figure S20. Stack-plot of ^{11}B NMR spectra of irradiated samples of **2-Dur** (■) in C_6D_6 (**2'-Dur** ♦).

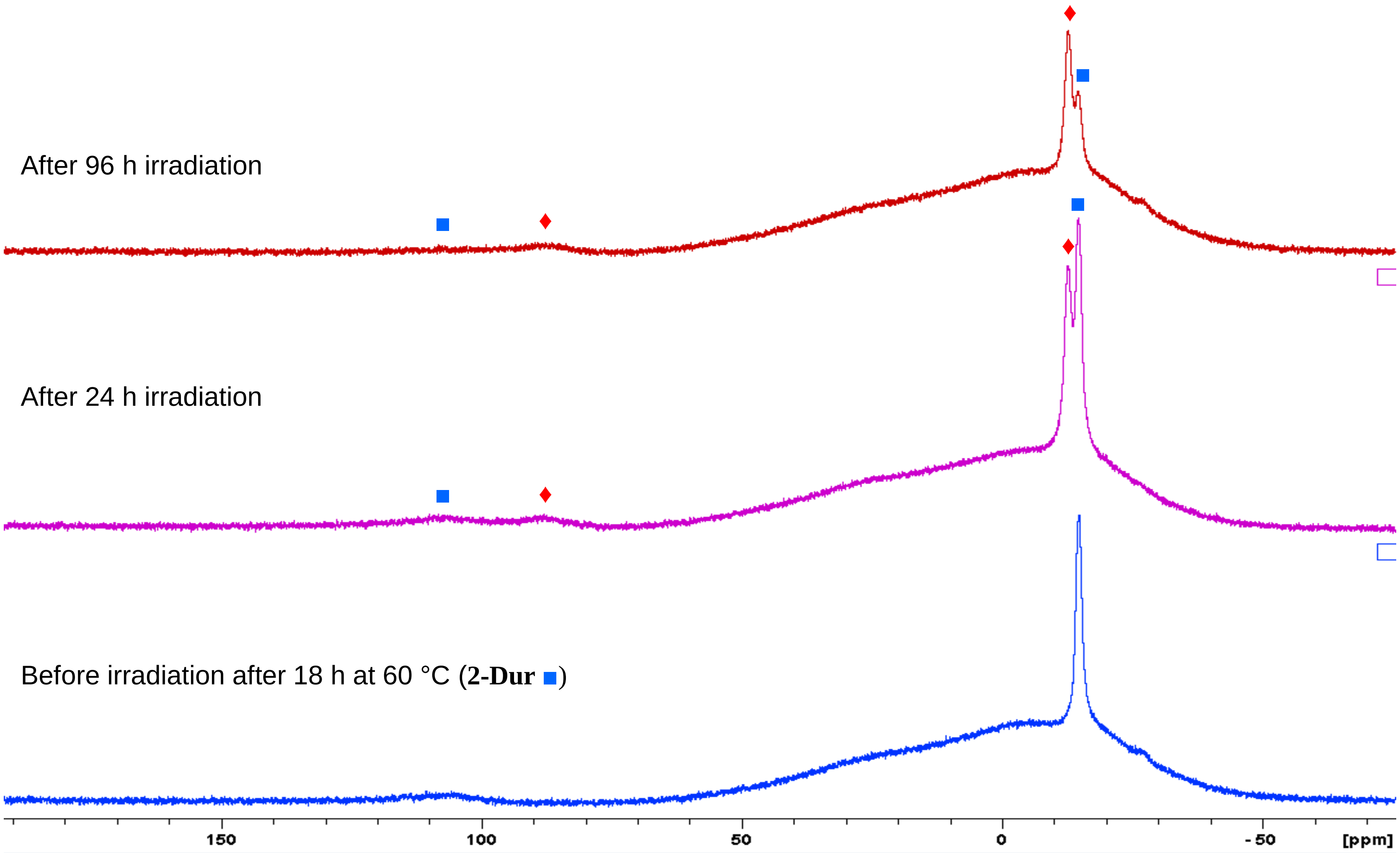

Figure S21. Stack-plot of $^{1}H\{^{11}B\}$ NMR spectra of irradiated samples of **2-Dur** (■) in C_6D_6 (**2'-Dur** ♦).

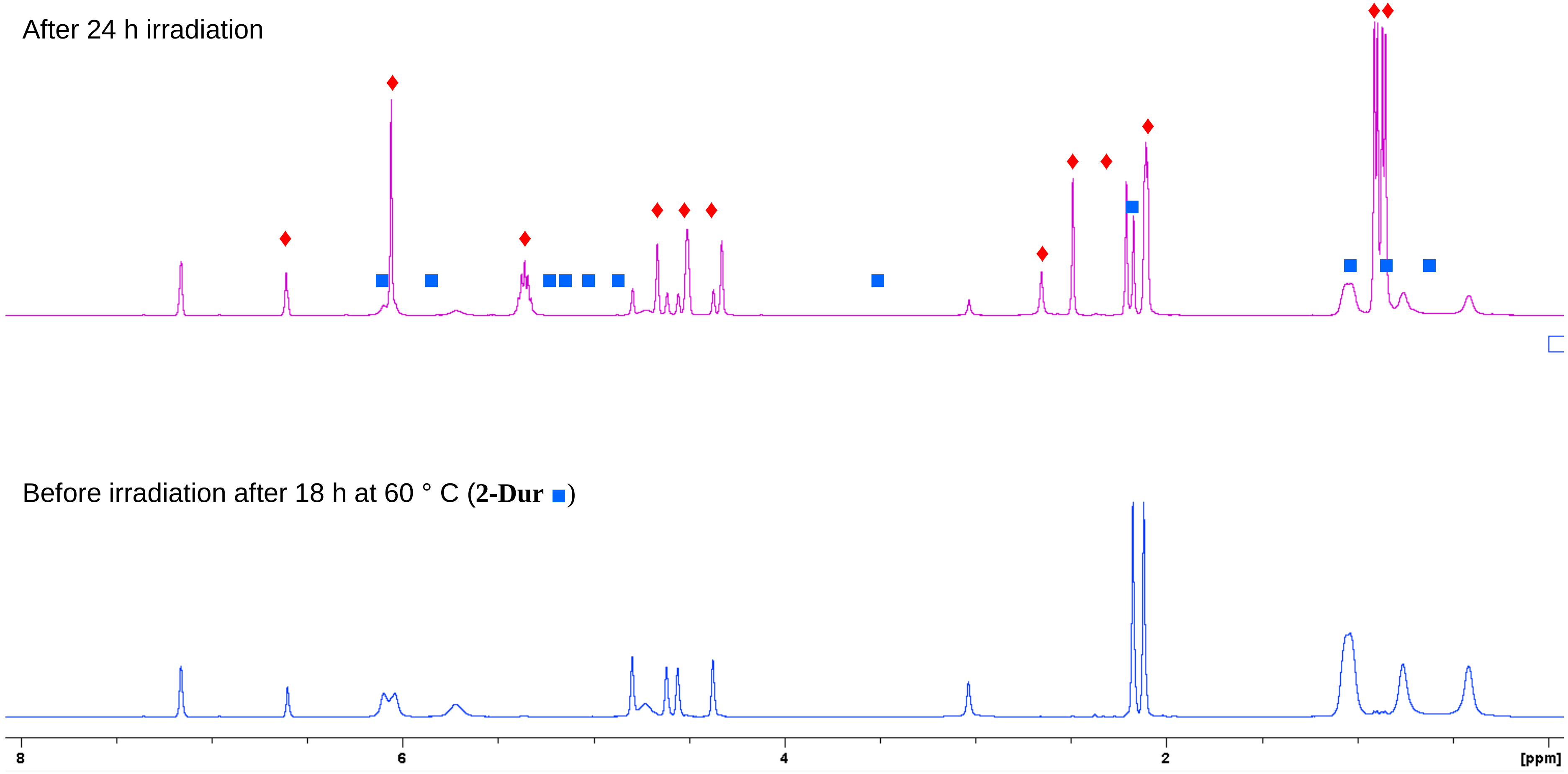

Kinetics of the thermal isomerization of 2'-Mes back to 2-Mes

Figure S22. Stackplot of ^{1}H NMR spectra of the isomerization reaction from **2'-Ar** to **2-Ar** in C_6D_6 at 60 °C over a period of 3 hours.

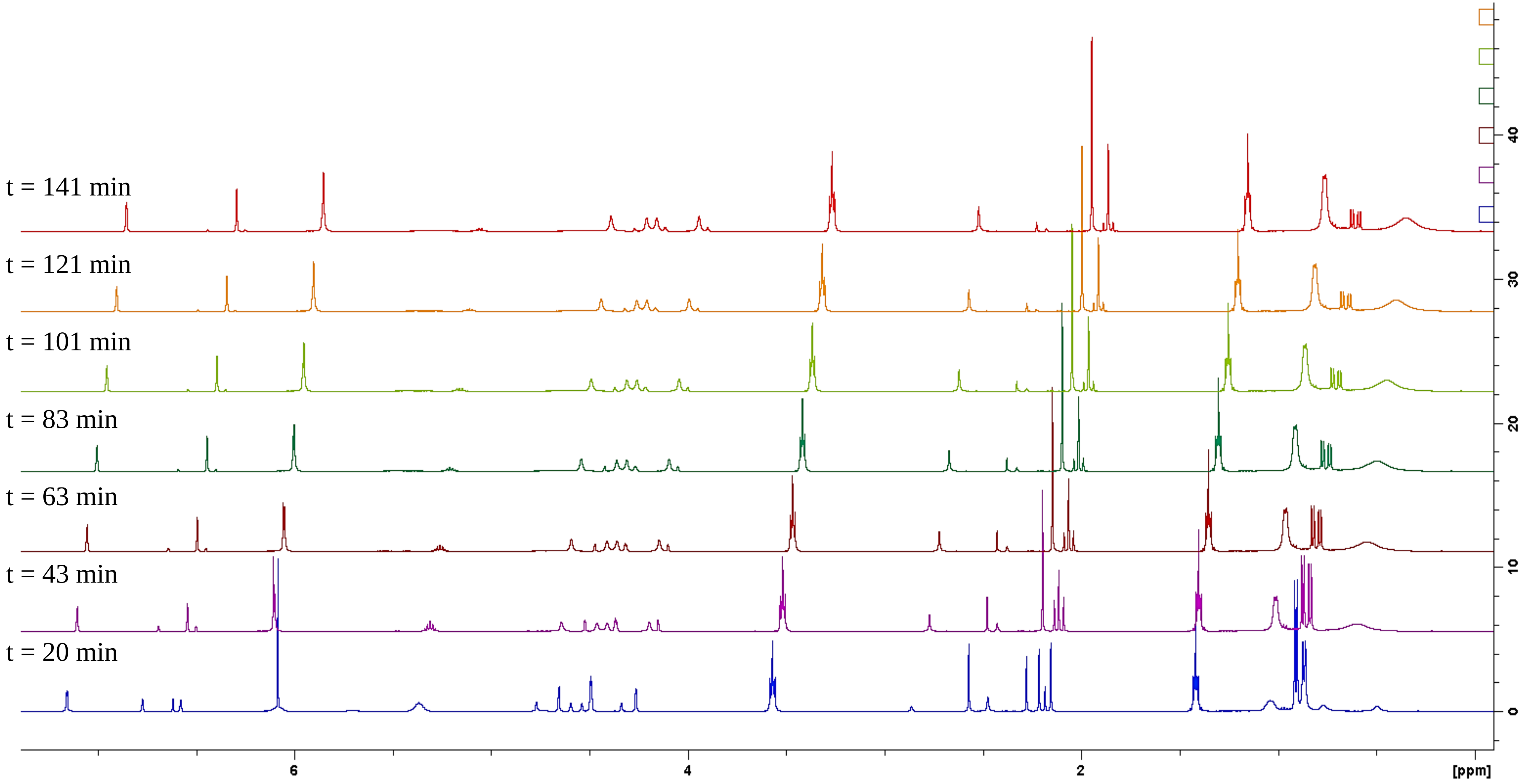

Figure S23. Plot of [**2'-Mes**] versus time in C_6D_6 at 60 °C over a period of 3 hours.

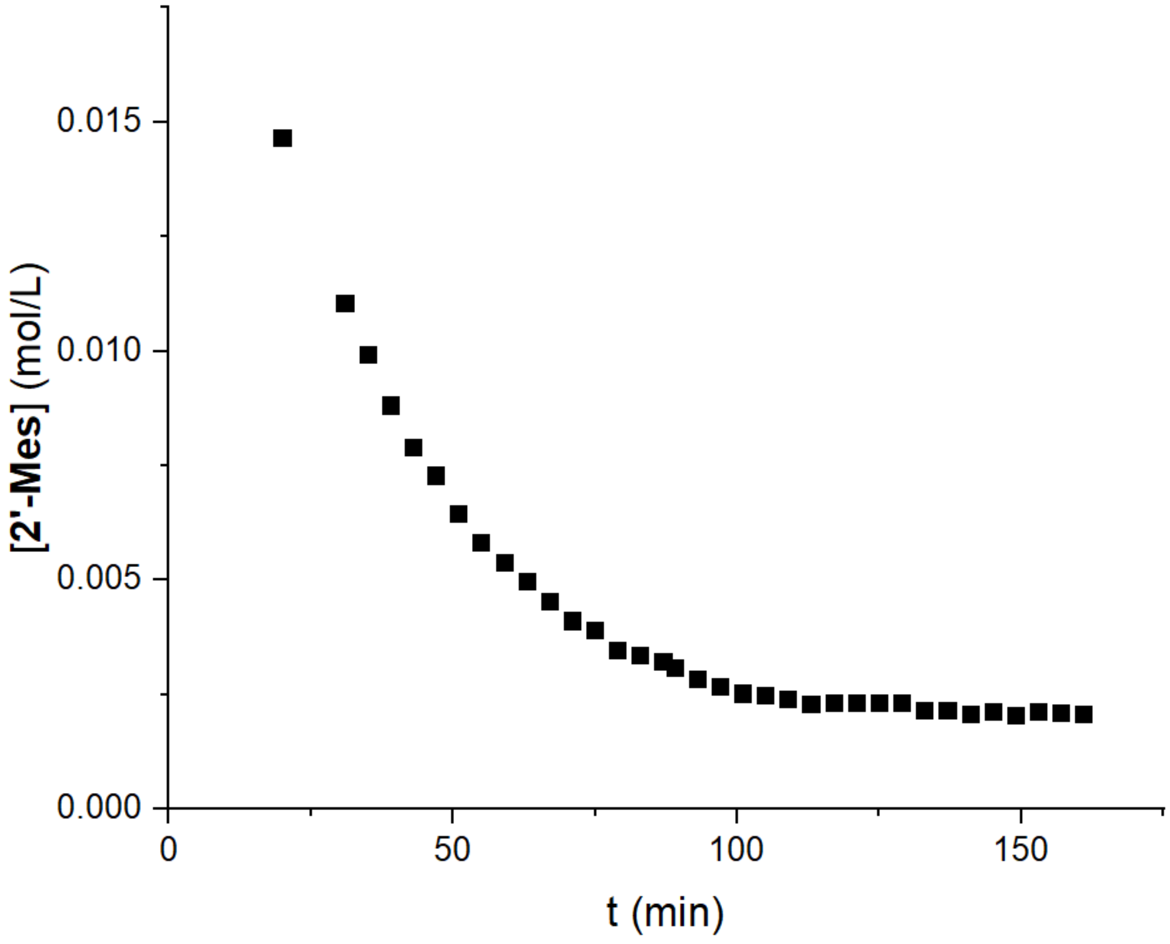

Figure S24. Left: plot of –ln[**2'-Ar**] versus t and linear fitting to a 1st-order rate plot. Right: plot of 1/√[2'-Ar] versus t and linear fitting to a half-order rate plot.

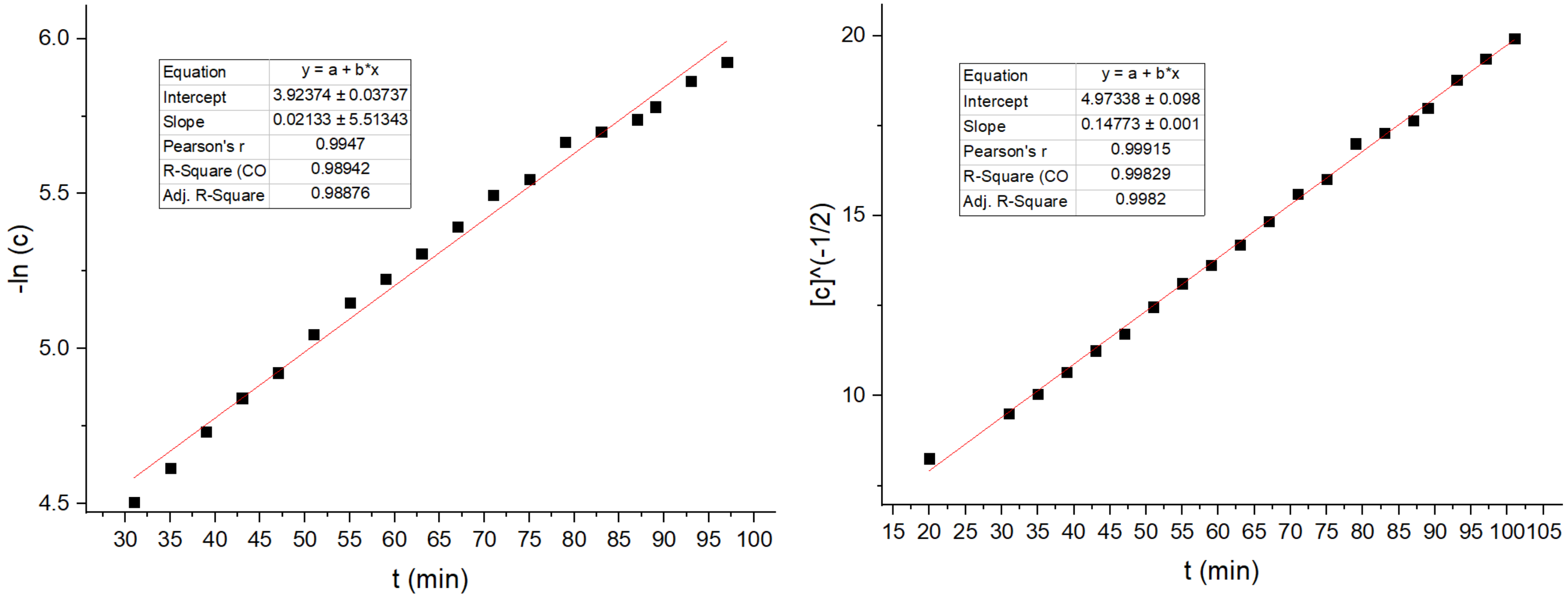

Cationization of 2-Pyr

Figure S25. Stack-plot of ^{11}B NMR spectra of freshly isolated crude **3-Pyr** in CD_2Cl_2 (top) and after several days at rt (bottom)

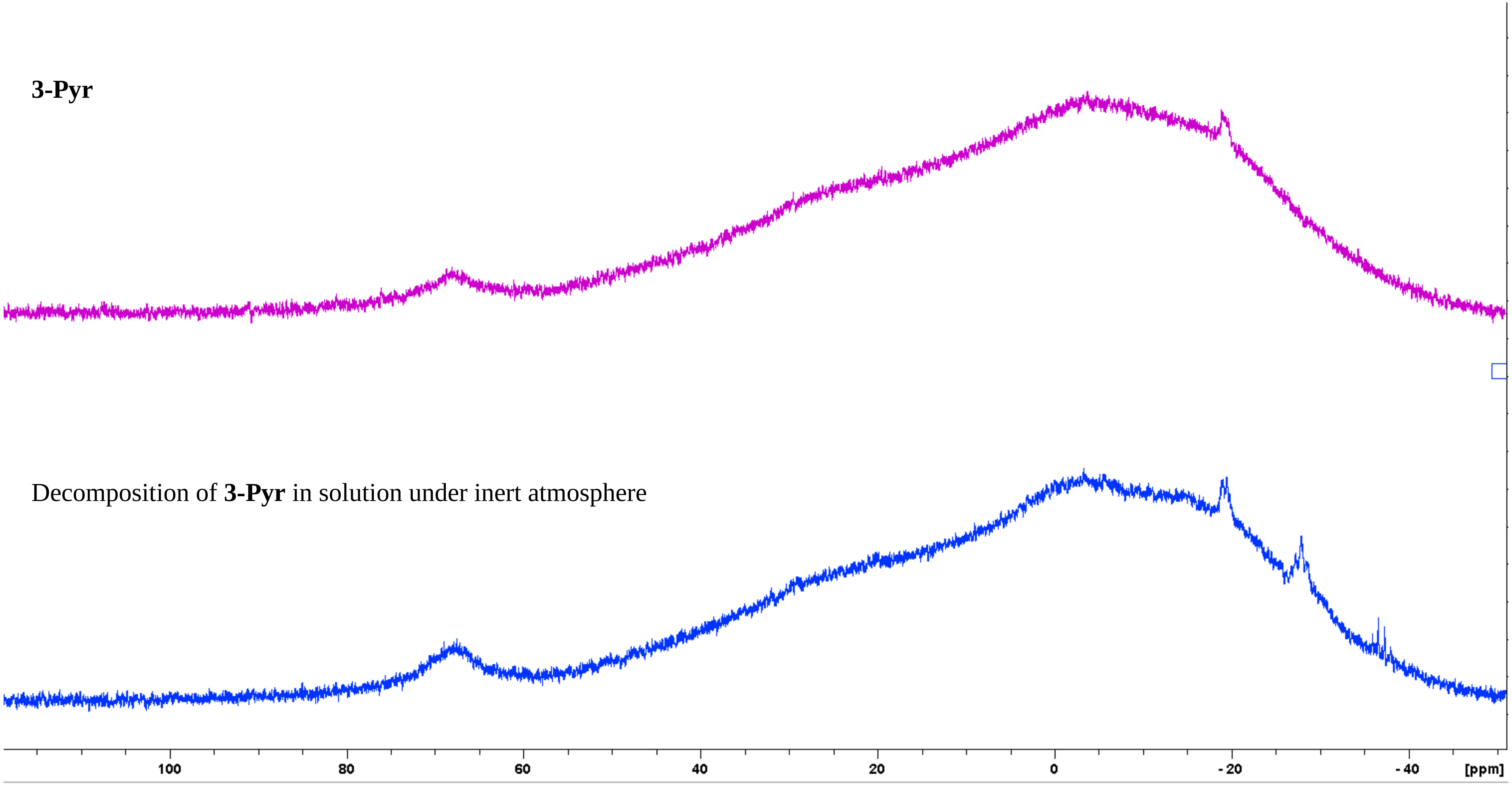

IR spectra

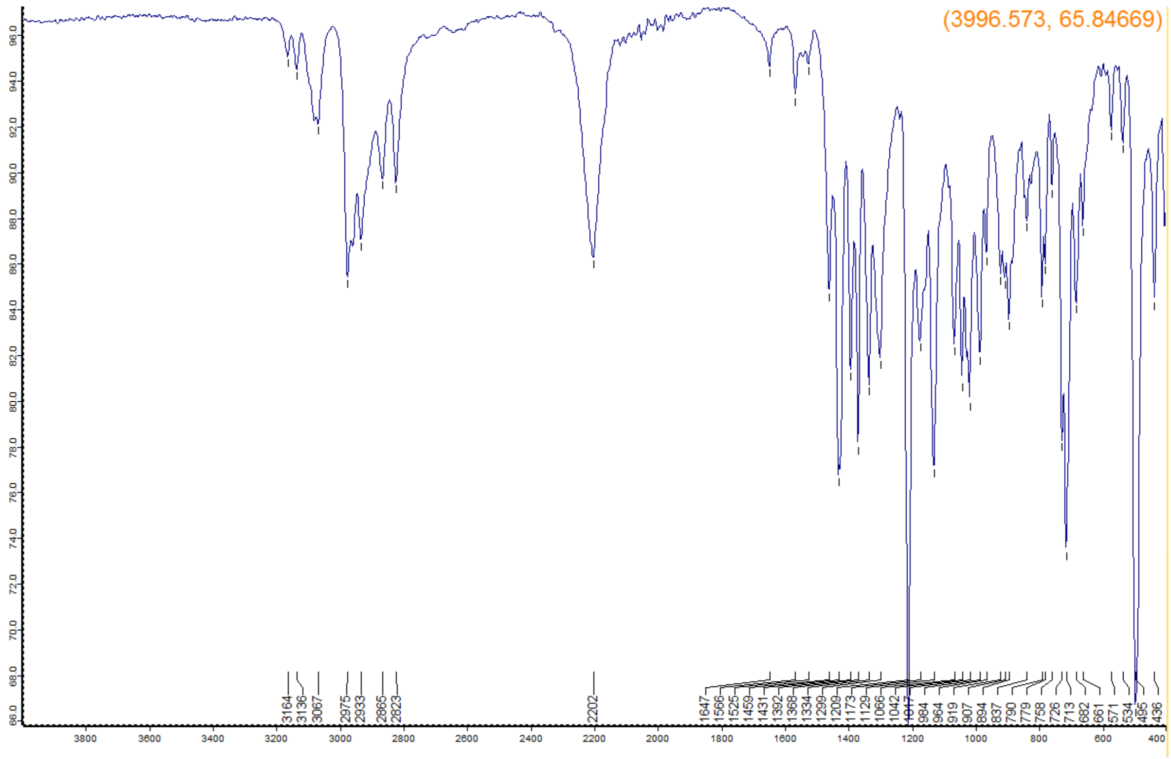

Figure S26. Solid-state IR spectrum of **2-Pyr.**

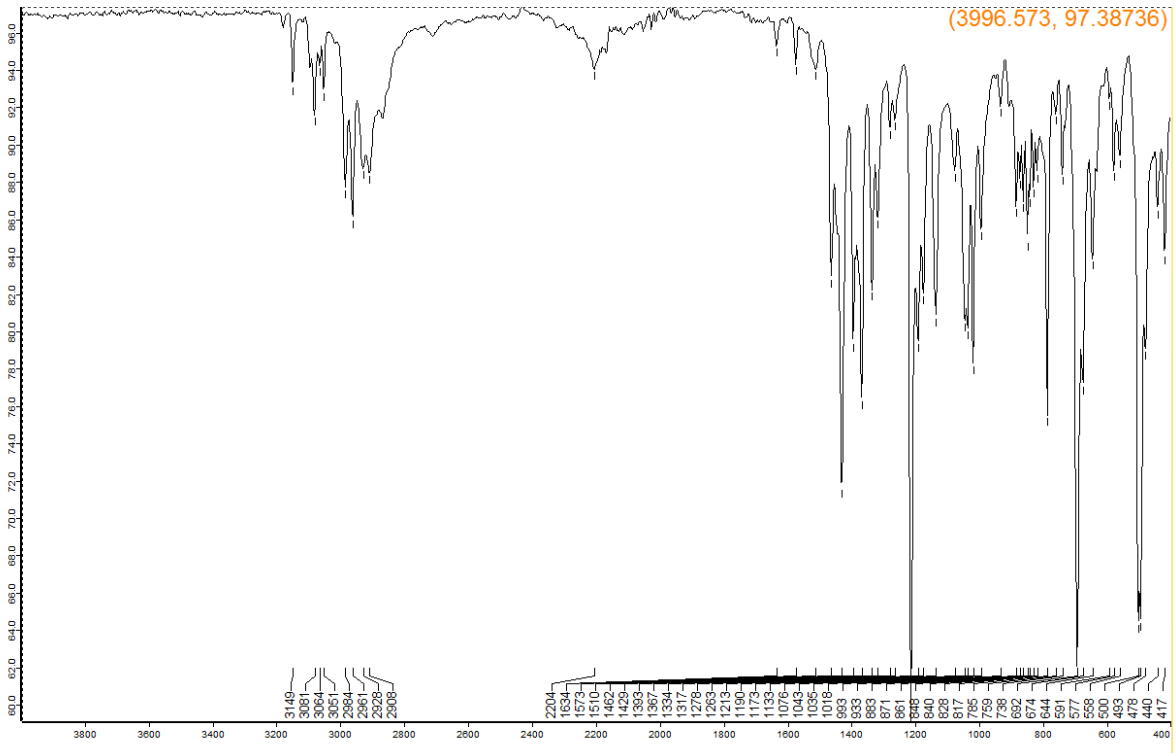

Figure S27. Solid-state IR spectrum of **2-Mes.**

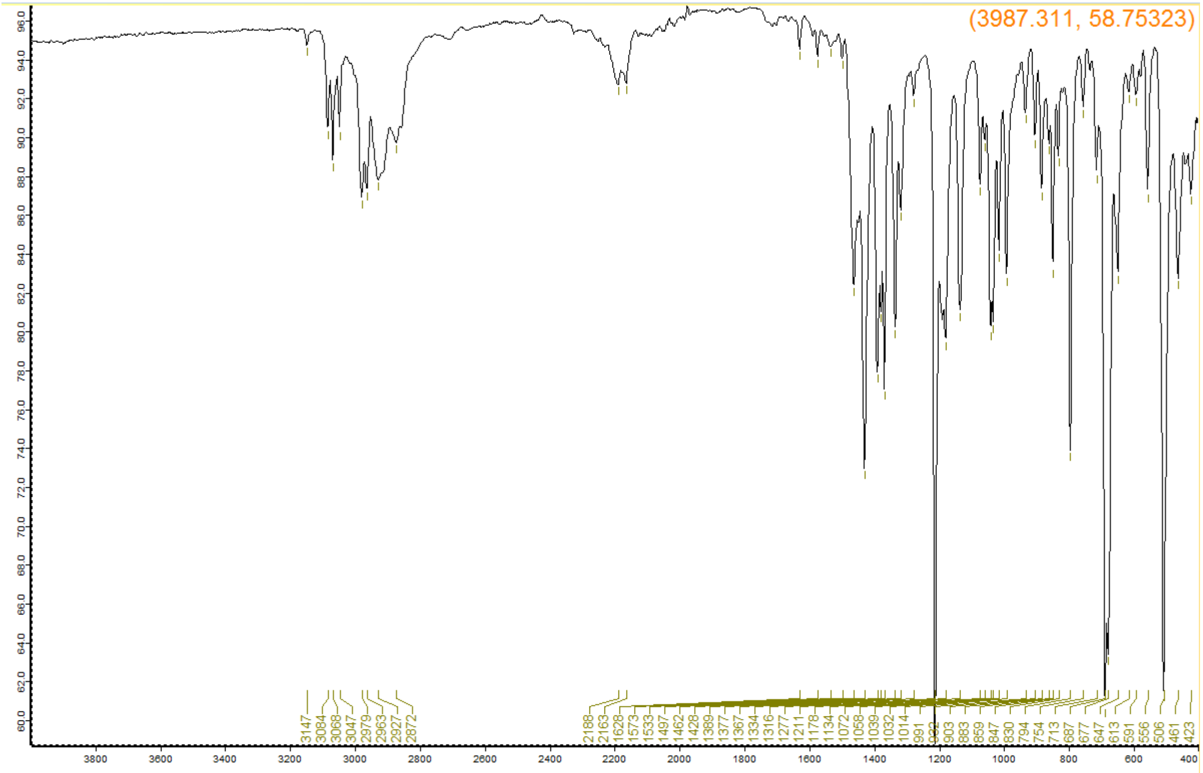

Figure S28. Solid-state IR spectrum of **2-Dur.**

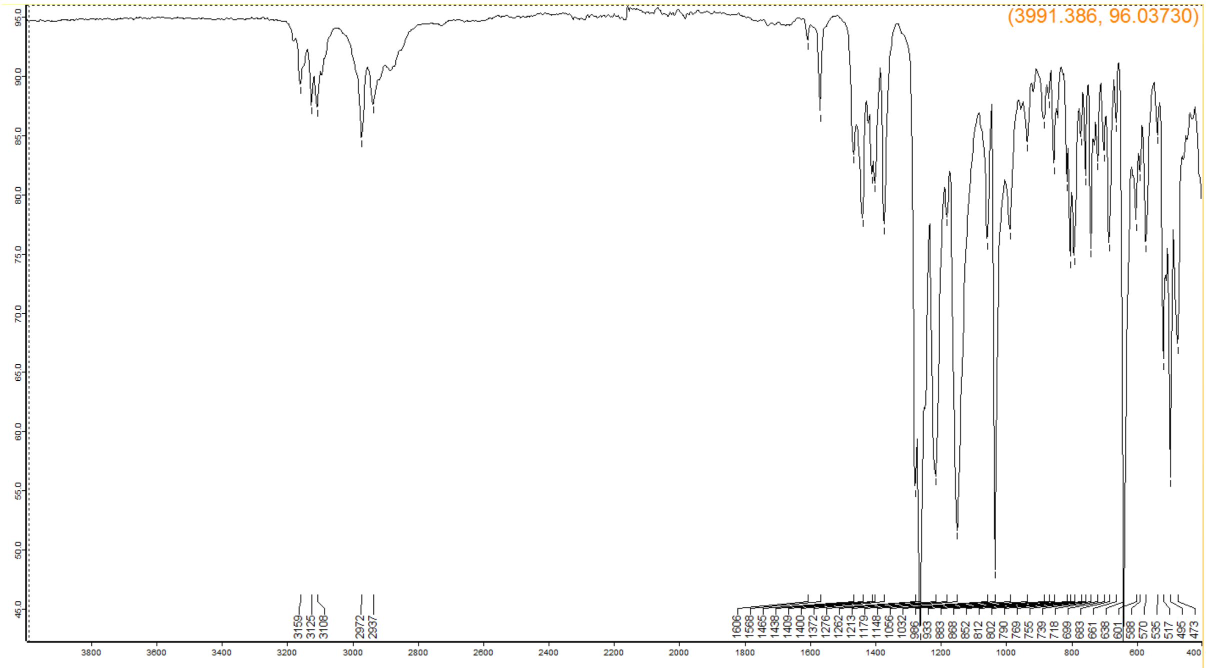

Figure S29. Solid-state IR spectrum of **3-Mes.**

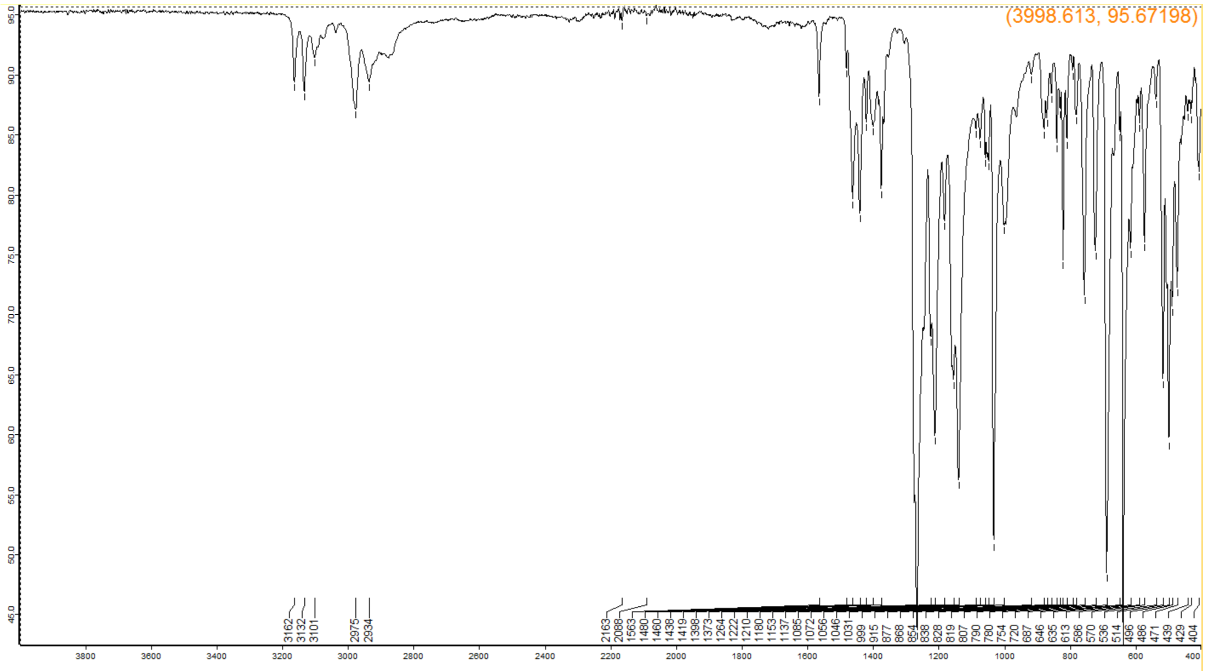

Figure S30. Solid-state IR spectrum of **3-Dur.**

X-ray crystallographic data

The crystal data of all compounds were collected on a Bruker X8-APEX II diffractometer with a CCD area detector (**2-Mes**, **2-Dur**, **2'-Mes**) or a BRUKER D8 QUEST diffractometer with a CMOS area detector (**2-Pyr**, **2'-Dur**, **3-Mes**), each equipped with *m*-layer mirror monochromated $Mo_{K\alpha}$ radiation. The structures were solved using intrinsic phasing methods,[3] refined with the ShelXL[4] software package and expanded using Fourier techniques. All non-hydrogen atoms were refined anisotropically. Hydrogen atoms were refined isotropically and assigned to idealized positions, except for boron-bound hydrogen atoms, which were located in the difference Fourier map and freely refined.

Cif files of crystallographic structures have been deposited with the Cambridge Crystallographic Data Center under the CCDC numbers 1947884 (**2-Pyr**), 1947885 (**2'-Dur**), 1947886 (**2-Dur**), 1947887 (**3-Dur**), 1947888 (**3-Mes**), 1947889 (**2-Mes**) and 1947890 (**2'-Mes**).

Crystal data for **2-Mes**: $C_{37}H_{53}B_3FeN_4$, M_r = 642.11, yellow block, 0.535×0.351×0.13 mm^3, monoclinic space group $P2_1/n$, a = 11.647(7) Å, b = 15.707(10) Å, c = 19.858(15) Å, β = 93.26(2)°, V = 3627(4) $Å^3$, Z = 4, ρ_{calcd} = 1.176 g·cm^{-3}, μ = 0.446 mm^{-1}, F(000) = 1376, T = 100(2) K, R_1 = 0.0395, wR^2 = 0.0975, 7147 independent reflections [$2\theta \leq 52.038°$] and 425 parameters.

Refinement details for **2-Dur**: The structure is C2-symmetric, the symmetry axis passing via Fe1 and B2. This generates a 1:1 disorder in the duryl moiety, for which the ADPs have been restrained with SIMU 0.001 and the ring modelled with AFIX 66.

Crystal data for **2-Dur**: $C_{38}H_{55}B_3FeN_4$, M_r = 656.14, orange block, 0.239×0.207×0.145 mm^3, monoclinic space group $C2/c$, a = 14.947(4) Å, b = 12.846(4) Å, c = 19.980(3) Å, β = 108.298(12)°, V = 3642.2(15) $Å^3$, Z = 4, ρ_{calcd} = 1.197 g·cm^{-3}, μ = 0.446 mm^{-1}, F(000) = 1408, T = 100(2) K, R_1 = 0.0405, wR^2 = 0.0925, 4517 independent reflections [$2\theta \leq 56.592°$] and 254 parameters.

Refinement details for **2-Pyr**: Four reflections affected by the beamstop were omitted. The molecule is C2-symmetric, the symmetry axis passing via Fe1, B2 and N3. ADPs in the 1:1 disordered central pyrrolidinyl fragment (RESI 3 pyrr, C15 > C18) were restrained with SIMU 0.005. C-C bond lengths within this residue were equalized with SADI. The molecule also presents a twofold disorder in one *i*Pr fragment (C13, C14, RESI 2 and 22) by rotation around N2-C12, modelled with FVAR in a 54:46 ratio, and with ADPs restrained with SIMU 0.003. The asymmetric unit contains one THF molecule which was modelled as threefold disordered, the ratio of the three parts (RESI 1, 11 and 111) being optimized with SUMP 1.0 to 54:26:20. Bond lengths were restrained to be similar in all three parts using SAME. ADPs were restrained using SIMU 0.003.

Crystal data for **2-Pyr**: $C_{32}H_{50}B_3FeN_5\cdot(C_4H_8O)_2$, $M_r = 737.25$, yellow block, 0.321×0.311×0.286 mm³, monoclinic space group *C*2/*c*, $a = 16.163(4)$ Å, $b = 11.923(3)$ Å, $c = 20.722(5)$ Å, $\beta = 93.281(16)°$, $V = 3986.8(17)$ Å³, $Z = 4$, $\rho_{calcd} = 1.228$ g·cm^{-3}, $\mu = 0.419$ mm^{-1}, $F(000) = 1592$, $T = 100(2)$ K, $R_1 = 0.0588$, $wR^2 = 0.1351$, 4242 independent reflections [2θ≤53.624°] and 368 parameters.

Refinement details for **2'-Mes**: The asymmetric unit contains 1.5 molecules of benzene (RESI Bz), one twofold disordered in a 67:33 ratio. ADPs of these parts were restrained with SIMU 0.01. The second half benzene molecule lies on an inversion center.

Crystal data for **2'-Mes**: $C_{37}H_{53}B_3FeN_4\cdot(C_6H_6)_{1.5}$, $M_r = 759.27$, yellow block, 0.251×0.203×0.14 mm³, monoclinic space group $P2_1/n$, $a = 13.0229(8)$ Å, $b = 18.2219(11)$ Å, $c = 18.0696(11)$ Å, $\beta = 101.9820(19)°$, $V = 4194.5(4)$ Å³, $Z = 4$, $\rho_{calcd} = 1.202$ g·cm^{-3}, $\mu = 0.396$ mm^{-1}, $F(000) = 1628$, $T = 100(2)$ K, $R_1 = 0.0391$, $wR^2 = 0.0942$, 8570 independent reflections [2θ≤52.74°] and 561 parameters.

Refinement details for **2'-Dur**: Data completeness was not reached (98.9% at 0.85 Å) due to very poor diffraction. The atom connectivity was, however, unambiguously assigned and the

boron-bound hydrogens located in the Fourier difference map. Due to the poor data quality SIMU restraints were applied on most carbon atoms.

Crystal data for **2'-Dur**: $C_{38}H_{55}B_3N_4Fe\cdot(C_6H_6)$, M_r = 734.27, orange plate, 0.36×0.319×0.177 mm^3, triclinic space group $P\bar{1}$, a = 10.450(4) Å, b = 11.632(4) Å, c = 17.599(8) Å, α = 77.32(2)°, β = 80.753(15)°, γ = 87.466(14)°, V = 2059.9(14) Å^3, Z = 2, ρ_{calcd} = 1.184 g·cm^{-3}, μ = 0.401 mm^{-1}, F(000) = 788, T = 100(2) K, R_1 = 0.1466, wR^2 = 0.1443, 6949 independent reflections [2θ≤49.426°] and 489 parameters.

Crystal data for **3-Mes**: $C_{38}H_{52}B_3F_3FeN_4O_3S$, M_r = 790.17, red block, 0.258×0.174×0.135 mm^3, orthorhombic space group $P2_12_12_1$, a = 10.854(3) Å, b = 16.233(2) Å, c = 22.903(7) Å, V = 4035.4(18) Å^3, Z = 4, ρ_{calcd} = 1.301 g·cm^{-3}, μ = 0.480 mm^{-1}, F(000) = 1664, T = 100(2) K, R_1 = 0.0315, wR^2 = 0.0621, 7937 independent reflections [2θ≤52.038°] and 492 parameters.

Refinement details for **3-Dur**: The asymmetric unit contains two half benzene molecules positioned on inversion centers. Although Platon[5] suggests a higher symmetry for the asymmetric unit this does not improve the data refinement.

Crystal data for **3-Dur**: $C_{39}H_{54}B_3F_3FeN_4O_3S\cdot(C_6H_6)$, M_r = 882.31, yellow needle, 0.733×0.434×0.064 mm^3, monoclinic space group $P2_1/n$, a = 15.4678(13) Å, b = 15.4962(14) Å, c = 19.4903(18) Å, β = 100.706(3)°, V = 4590.4(7) Å^3, Z = 4, ρ_{calcd} = 1.277 g·cm^{-3}, μ = 0.429 mm^{-1}, F(000) = 1864, T = 100(2) K, R_1 = 0.0357, wR^2 = 0.0808, 9032 independent reflections [2θ≤52.038°] and 557 parameters.

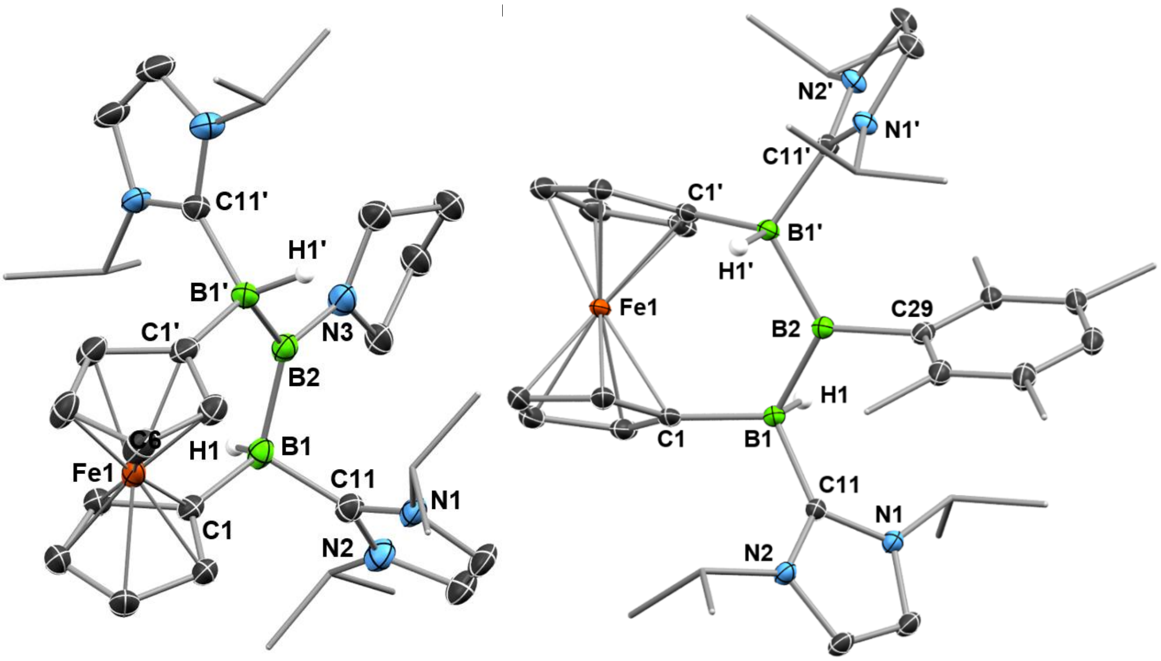

Figure S31. Crystallographically derived molecular structures of **2-Pyr** (left) and **2-Dur** (right). Atomic displacement ellipsoids are set at 50% probability. Ellipsoids of *iso*propyl groups and hydrogen atoms omitted for clarity except for boron-bound hydrides.

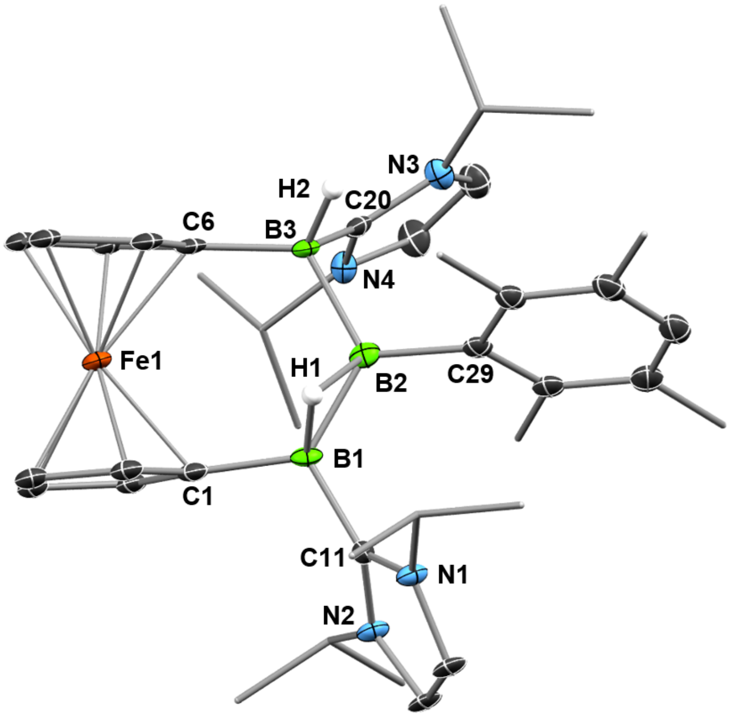

Figure S32. Crystallographically derived molecular structure of **2'-Dur**. Atomic displacement ellipsoids are set at 50% probability. Ellipsoids of *iso*propyl groups and hydrogen atoms omitted for clarity except for boron-bound hydrides.

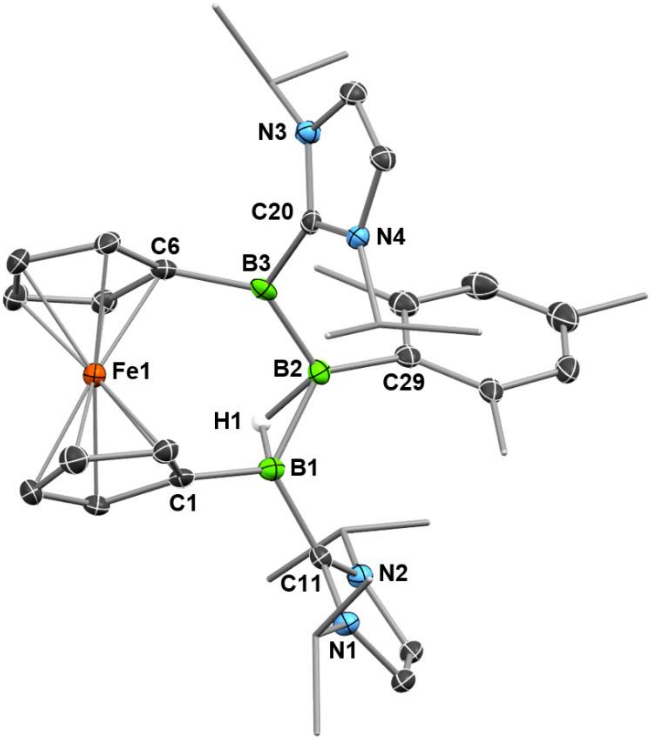

Figure S33. Crystallographically derived molecular structure of the **3-Mes** cation. Atomic displacement ellipsoids are set at 50% probability. Ellipsoids of methyl and *iso*propyl groups, the triflate anion and hydrogen atoms omitted for clarity except for boron-bound hydrides.

Table S1. Relevant bond lengths (Å) and angles (°) for **2-Pyr**, **2-Mes**, **2-Dur**, **2'-Mes**, **3-Mes** and **3-Dur**.

	**2-Pyr**	**2-Mes**	**2-Dur**	**2'-Mes**	**3-Mes**	**3-Dur**
B1-B2	1.756(4)	1.720(3)	1.724(2)	1.650(3)	1.666(3)	1.658(2)
B2-B3	–	1.738(3)	–	1.774(3)	1.667(4)	1.667(2)
B1-H1	1.14(3)	1.24(3)	1.15(2)	1.22(2)	1.25(3)	1.266(16)
B2-H1	–	–	–	1.50(2)	1.36(2)	1.338(17)
B3-H2	–	1.13(2)	–	–	–	–
B1-C1	1.612(4)	1.598(3)	1.613(2)	1.598(3)	1.595(3)	1.590(2)
B3-C6	–	1.611(3)	–	1.621(3)	1.548(4)	1.545(2)
B1-C11	1.617(4)	1.616(3)	1.615(2)	1.601(3)	1.594(3)	1.596(2)
B3-C20	–	1.623(3)	–	1.616(3)	1.601(3)	1.601(2)
B2-X[a]	1.413(5)	1.590(3)	1.632(3)	1.599(3)	1.607(3)	1.600(2)
B1-B2-B3[b]	127.0(3)	118.72(17)	119.78(17)	122.69(16)	111.3(2)	110.49(12)
Tilt angle Cp/Cp α	2.3	7.7	7.3	6.1	6.7	5.6
Dihedral angle B1,C1,C6,B3[c]	6.8	37.2	32.4	9.9	33.1	33.7

[a] X = N3 for **2-Pyr**, C29 for Mes and Dur derivatives; B1-B2-B1' for **2-Pyr** and **2-Dur**.

[b] B1,C1,C1',B1' for **2-Pyr** and **2-Dur**.

Computations

Geometry optimizations and EDA analysis were carried out using the Amsterdam Density Functional (ADF)[6,7] program at the OLYP/ZORA/TZ2P[8-12] level of theory. To obtain the singlet state, spin-restricted calculations were performed constraining the projection of the total electronic spin along a reference axis to 0. Frequency calculations were conducted to determine if each stationary point corresponds to a minimum.[13-15] Reported bond orders are of the Mayer bond order type[16,17] and atomic charges were determined according to the Hirshfeld charge analysis.[18,19] ^{11}B NMR shifts were calculated with the GIAO method using the corresponding BF_3OEt_2 shielding at the same level of theory as reference.[20,21] The Graphical User Interface (ADF-GUI) – a part of the ADF package – was used for visualization purposes.

On the **2-Mes** to **2'-Mes** tautomerism.

As stated in the main text, excited state optimization of **2-Mes** forces the system to become the *anti*-analogue of the **2'-Mes** isomer, i.e. with one bridging H and one terminal H in a mutually *anti* conformation (see Figure S26). Closer examination of the MOs of *anti*-**2'-Mes** suggests that on two adjacent borons in the B3 unit, two in-phase p orbitals, each positioned on a boron center, are tilted in such a way that the 'upper' lobes are effectively interacting, on the other side of the nodal plane, while the two other lobes are interacting with the bridging hydrogen atom, and the p orbital on the third boron is interacting with the other hydrogen (see Figure S27). Upon excitation the system moves from the σ(B1(p)-B2(p)-B3(p)) framework of the B3 core (see Figure S28), in which the two hydrogens are perpendicular to the B3 axis, into a π*(B1(p)-B2(p)-B3(p) configuration. The latter evolves into the theoretically calculated *anti*-**2'-Mes** isomer. At this point one can conjecture that further excitation brings about the migration of the bridging hydrogen towards a *syn* conformation. This can be ascribed to the more fundamental concept of the evolution over time of the superposition of states, hence the role of the phase of the wavefunction, i.e. the B-H-B bridge structure can acquire antibonding character. Secondly, upon going from the *trans*-**2-Mes** geometry towards *trans*-**2'-Mes**, one can look for instance at the correlation between the B1(extremal)-H(bridging) bond length and the total energy of the system (see Figure S30). There is a quasi-instantaneous (vertical) increase in system energy, which then stabilizes in a plateau over longer times in the phase space. On the other hand, the B-H distance only gradually increases up to a point where the total energy appears to be entirely a function of this particular bond length. One could

tentatively ascribe the jump in energy to some conrotatory 'motion' of the σ(B1(p)-B2(p)-B3(p) framework leading ultimately to favoring the interaction of the (about-to-be-bridging) hydrogen with B2, hence to the progressive formation of the bridged species. In the optimized *anti*-**2'-Mes**, the LUMO shows an in-phase relation between the bridging hydrogen and the lobe of p(B1) that is *syn* with respect to the other hydrogen (see Fig. S31), in line with a possible migration upon further excitation, leading to the formation of *syn*-**2'-Mes**. A basic first-order transition state within the quasi-Newton method for the thermal **2'-Ar** to **2-Ar** path could not be found. We think that this observation cannot be attributed to algorithm issues (e.g. stronger anharmonicities at the TS). Rather, this may be in line with the unusual half-order rate pointing to a more complex transformation.

The apparent exchange of bridging and terminal hydrides of **2'-Ar** in solution implied by the apparent symmetry in their ^{1}H and ^{11}B NMR spectra was also investigated computationally. However, no convenient structures (minima, transition states, higher multiplicities, ionised) were found that are consistent with a hydrogen terminal/bridging fluxionality within a given close- or open-shell PES. Calculated structures with non-bridging atoms perpendicular to the B_3 plane (valid for *cis*- and *trans*-isomers) are either a ground state or a singlet excited state. Further studies using deformation densities suggest that there is a dynamic structure-dependent charge flow between two outer B-H units but this is not conclusive pertaining to a given exchange. As suggested above this may be indicative of a more complex mechanism.

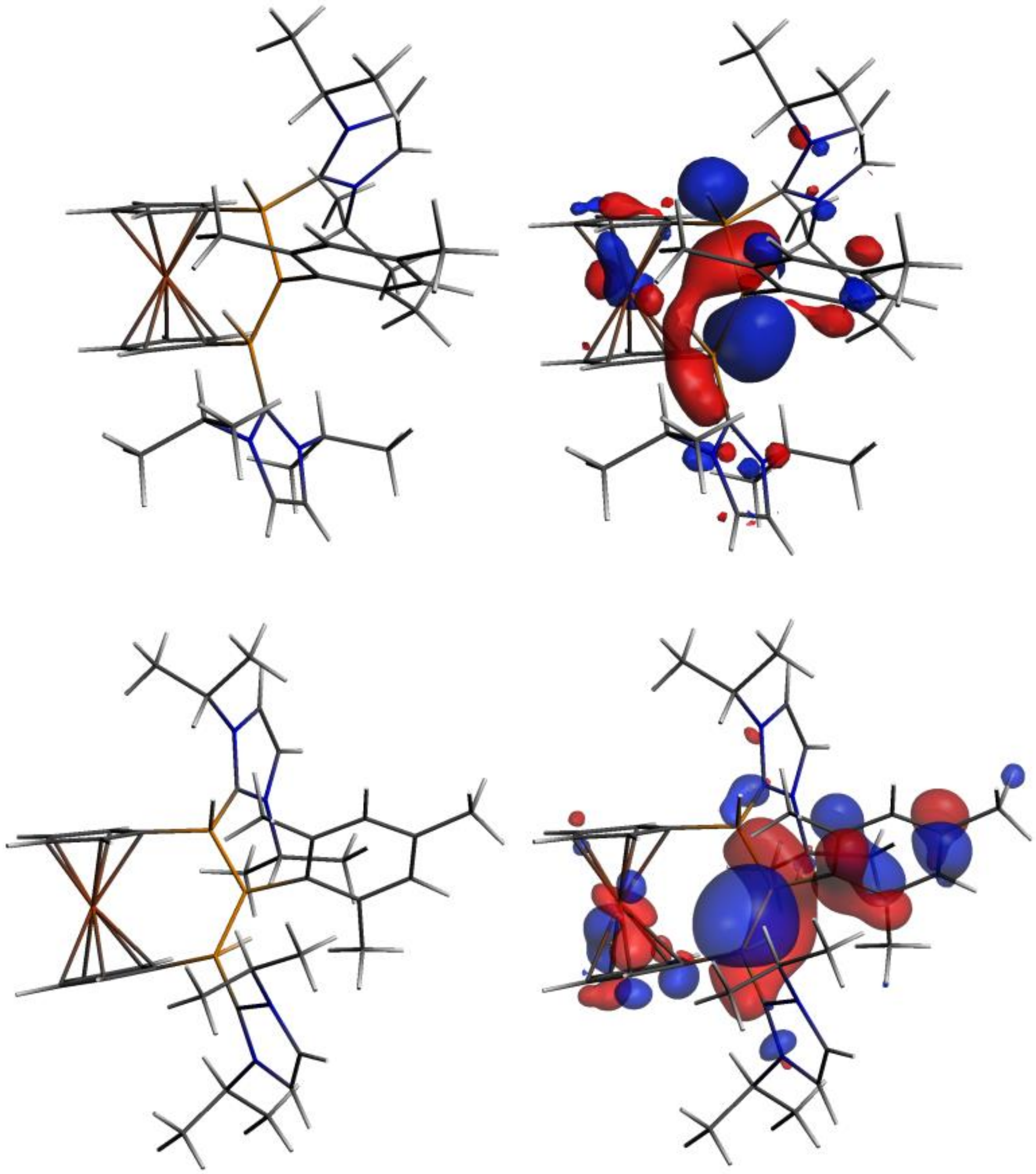

Figure S34. Calculated structure and HOMO-3 of **2'-Mes** (top) and *anti*-**2'-Mes** (bottom) obtained as the optimized excited state of **2-Mes**.

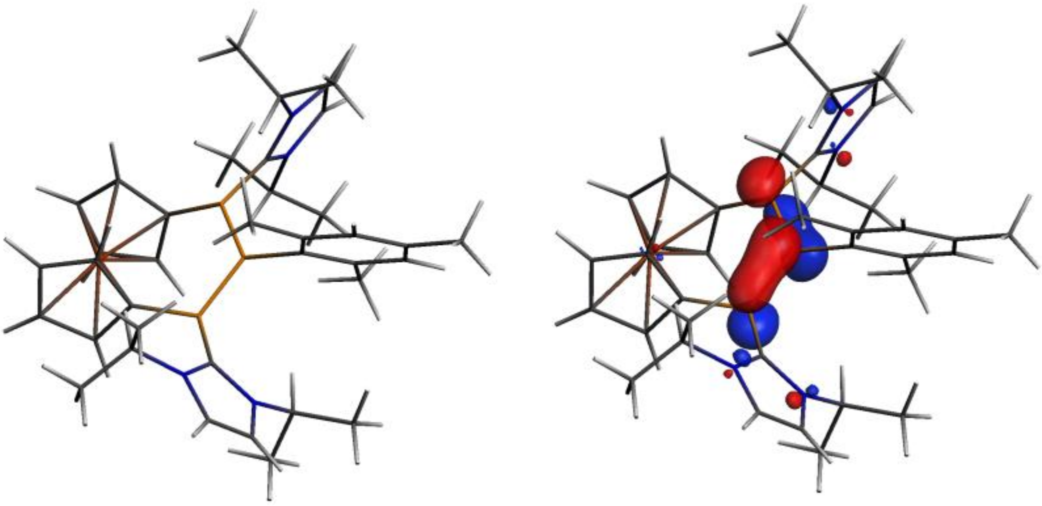

Figure S35. Calculated structure and HOMO of **2-Mes***, a hypothetical neutral singlet system, in which the two boron-bound hydrogen atoms have been abstracted. The HOMO represents the π orbital delocalized over the B_3 system and displays out-of-phase spherical lobe remnants of a p orbital at B1 and B3 pointing in opposite directions.

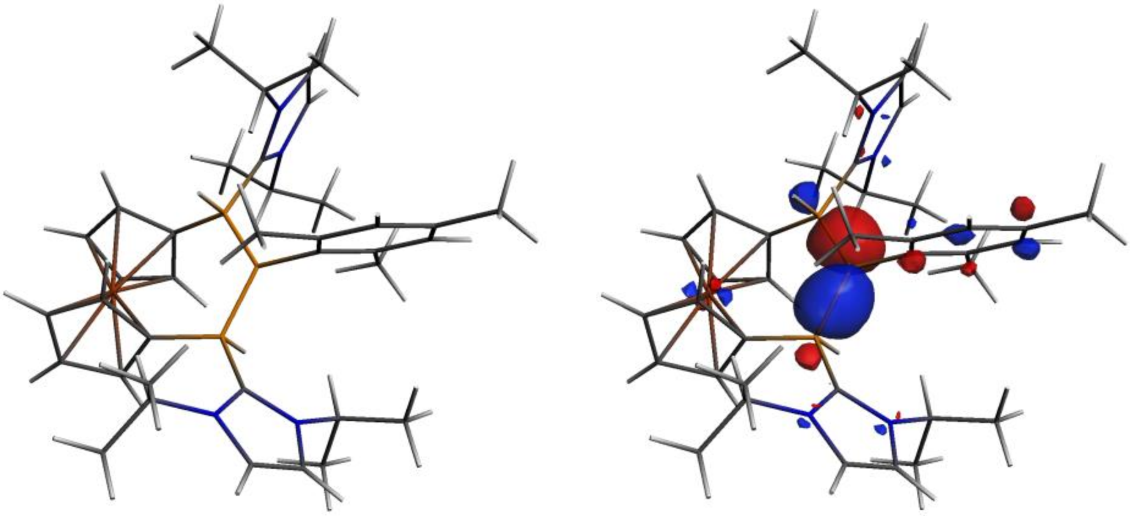

Figure S36. Calculated structure and HOMO-3 of **2-Mes**.

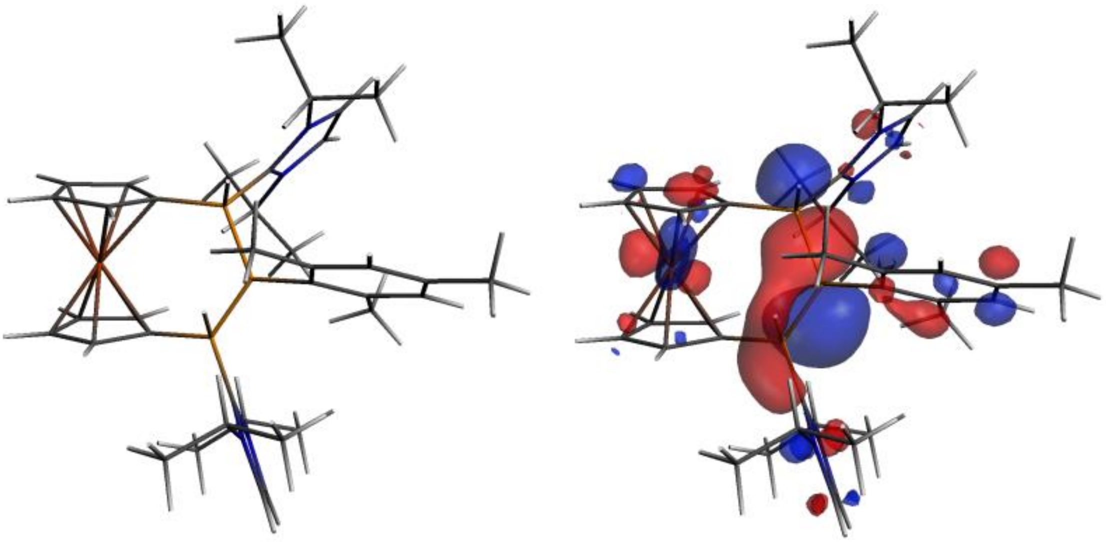

Figure S37. Calculated structure and HOMO-3 of the hypothetical *syn*-**2-Mes**.

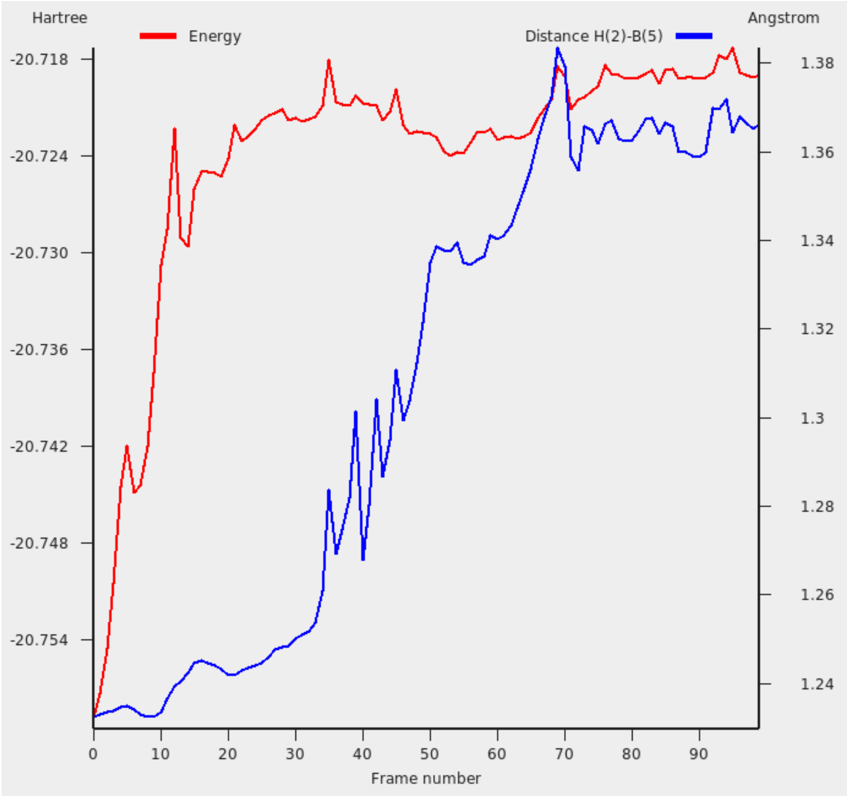

Figure S38. Overlay of the total electronic energy (red) and B1-H1 bond length upon excited state optimization of *trans*-**2-Mes**.

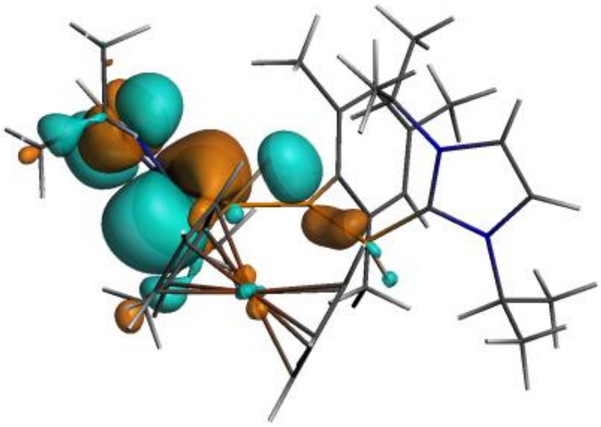

Figure S39. Calculated LUMO of the optimized excited state of *trans*-**2-Mes**.

References

[1] H. Braunschweig, I. Krummenacher, C. Lichtenberg, J. D. Mattock, M. Schäfer, U. Schmidt, C. Schneider, T. Steffenhagen, S. Ullrich, A. Vargas, *Angew. Chem. Int. Ed.* **2017**, 56, 889-892; *Angew. Chem*. **2017**, *129*, 907-911.

[2] N. Arnold, S. Mozo, U. Paul, U. Radius, H. Braunschweig, *Organometallics* **2015**, *34*, 5709-5715.

[3] G. Sheldrick, *Acta Cryst*. **2015**, *A71*, 3-8.

[4] G. Sheldrick, *Acta Cryst*. **2008**, *A64*, 112-122.

[5] A. L. Spek, *Acta Cryst.* **2009**, *D65*, 148-155.

[6] a) G. te Velde, F. M. Bickelhaupt, E. J. Baerends, C. Fonseca Guerra, S. J. A. van Gisbergen, J. G. Snijders, T. Ziegler, *J. Comput. Chem.* **2001**, *22*, 931-967; b) T. Ziegler, A. Rauk, *Theo. Chim. Acta*, **1977**, *46*, 1-10.

[7] Amsterdam Density Functional, Theoretical Chemistry, Vrije Universiteit, Amsterdam, The Netherlands, http://www.scm.com

[8] N. C. Handy, A. J. Cohen, *Mol. Phys.* **2001**, *99*, 403-412.

[9] D. P. Chong, *Mol. Phys.* **2005**, *103*, 749-761.

[10] D. P. Chong, E. van Lenthe, S. J. A. van Gisbergen, E. J. Baerends, *J. Comput. Chem.* **2004**, *25*, 1030-1036.

[11] E. van Lenthe, E. J. Baerends, *J. Comput. Chem.* **2003**, *24*, 1142-1156.

[12] R. C. Raffenetti, *J. Chem. Phys.* **1973**, *59*, 5936-5949.

[13] A. Berces, R. M. Dickson, L. Fan, H. Jacobsen, D. Swerhone, T. Ziegler, *Comput. Phys. Commun.* **1997**, *100*, 247-262.

[14] H. Jacobsen, A. Berces, D. Swerhone, T. Ziegler, *Comput. Phys. Commun.* **1997**, *100*, 263-276.

[15] S. K. Wolff, *Int. J. Quantum Chem.* **2005**, *104*, 645-659.

[16] I. Mayer, *Chem. Phys. Lett.* **1983**, *97*, 270-274.

[17] E. P. Fowe, B. Therrien, G. Süss-Fink, C. Daul, *Inorg. Chem.* **2008**, *47*, 42-48.

[18] F. L. Hirshfeld, *Theo. Chim. Acta*, **1993**, *44*, 129-138.

[19] K. B. Wiberg, P. R. Rablen, *J. Comp. Chem.* **1993**, *14*, 1504-1518.

[20] G. Schreckenbach, T. Ziegler, *Int. J. Quantum Chem.* **1996**, 60, 753-766.

[21] G. Schreckenbach, T. Ziegler, *Int. J. Quantum Chem.* **1997**, 61, 899-918.
